# PCPro: a clinically accessible, circulating lipid biomarker signature for poor-prognosis metastatic prostate cancer

**DOI:** 10.1038/s41391-023-00666-2

**Published:** 2023-05-05

**Authors:** Tahlia Scheinberg, Hui-Ming Lin, Michael Fitzpatrick, Arun A. Azad, Paul Bonnitcha, Amy Davies, Gillian Heller, Kevin Huynh, Blossom Mak, Kate Mahon, David Sullivan, Peter J. Meikle, Lisa G. Horvath

**Affiliations:** 1 Medical Oncology, Chris O’Brien Lifehouse, Camperdown, NSW Australia; 2 Advanced Prostate Cancer Group, Garvan Institute of Medical Research, Darlinghurst, NSW Australia; 3 University of Sydney, Camperdown, NSW Australia; 4 St Vincent’s Clinical School, UNSW Sydney, Sydney, NSW Australia; 5 NSW Health Pathology, Department of Chemical Pathology, Royal Prince Alfred Hospital, Camperdown, NSW 2050 Australia; 6 Department of Medical Oncology, Peter MacCallum Cancer Centre, Melbourne, VIC Australia; 7 Sir Peter MacCallum Department of Oncology, University of Melbourne, Melbourne, VIC Australia; 8 Department of Medical Oncology, Monash Health, Melbourne, VIC Australia; 9 Department of Medicine, Monash University, Melbourne, VIC Australia; 10 Baker Heart and Diabetes Institute, Melbourne, VIC Australia; 11 Department of Cardiovascular Research Translation and implementation, La Trobe University, Melbourne, VIC Australia; 12 Royal Prince Alfred Hospital, Camperdown, NSW Australia

**Keywords:** Cancer metabolism, Prostate cancer

## Abstract

**Background:**

Using comprehensive plasma lipidomic profiling from men with metastatic castration-resistant prostate cancer (mCRPC), we have previously identified a poor-prognostic lipid profile associated with shorter overall survival (OS). In order to translate this biomarker into the clinic, these men must be identifiable via a clinically accessible, regulatory-compliant assay.

**Methods:**

A single regulatory-compliant liquid chromatography-mass spectrometry assay of candidate lipids was developed and tested on a mCRPC Discovery cohort of 105 men. Various risk-score Cox regression prognostic models of OS were built using the Discovery cohort. The model with the highest concordance index (PCPro) was chosen for validation and tested on an independent Validation cohort of 183 men.

**Results:**

PCPro, the lipid biomarker, contains Cer(d18:1/18:0), Cer(d18:1/24:0), Cer(d18:1/24:1), triglycerides and total cholesterol. Within the Discovery and Validation cohorts, men who were PCPro positive had significantly shorter OS compared to those who were PCPro negative (Discovery: median OS 12.0 months vs 24.2 months, hazard ratio (HR) 3.75 [95% confidence interval (CI) 2.29–6.15], *p* < 0.001, Validation: median OS 13.0 months vs 25.7 months, HR = 2.13 [95% CI 1.46–3.12], *p* < 0.001).

**Conclusions:**

We have developed PCPro, a lipid biomarker assay capable of prospectively identifying men with mCRPC with a poor prognosis. Prospective clinical trials are required to determine if men who are PCPro positive will benefit from therapeutic agents targeting lipid metabolism.

## Introduction

Prostate cancer is the second most common cancer and fifth highest cause of cancer death in men worldwide [1]. Despite new treatments for metastatic castration-resistant prostate cancer (mCRPC), including taxane chemotherapy, androgen receptor signalling inhibitors (ARSI), poly-ADP ribose polymerase (PARP) inhibitors and lutetium-177-prostate-specific membrane antigen [2], men will either have intrinsic resistance or develop treatment resistance. New biomarkers and treatment strategies are needed. The long-term control of mCRPC requires strategies targeting multiple hallmarks of cancer including neoplastic cells, tumour microenvironment, inflammation, genetics and systemic metabolic factors (including lipid metabolism) [3].

Using high-throughput liquid chromatography-mass spectrometry (LC-MS) lipid profiling, we previously showed that baseline plasma lipidomic profiles enriched with sphingolipids such as ceramides and sphingomyelins are associated with shorter overall survival (OS) in men with mCRPC treated with docetaxel. The poor prognostic lipid profile could be represented by a three-lipid signature (3LS) (Ceramide(Cer)(d18:1/24:1), Sphingomyelin(SM)(d18:2/16:0) and Phosphatidylcholine(PC)(16:0/16:0)) [4]. Subsequent studies of mCRPC cohorts confirmed that presence of the 3LS before treatment with taxanes or ARSIs is associated with shorter OS [5–7]. Furthermore, elevated levels of circulating ceramides were associated with higher rates of metastatic relapse in localised PC [5].

Most circulating ceramides are derived from the liver [8] and are elevated in systemic inflammation [9]. However, prostate cancer cells also express the appropriate biosynthetic enzymes and may produce ceramides that are transported into the circulation [5, 10]. Ceramides may contribute to therapeutic resistance and tumour growth through their conversion into pro-survival sphingosine-1-phosphate (S1P) [8]. Signal transduction pathways mediated by S1P promote cancer cell proliferation, migration, invasion and regulate lymphocyte trafficking by acting on S1P-specific receptors present on immune cells and cancer cells [8]. Therefore, we hypothesise that a poor prognostic plasma lipid profile consisting of elevated sphingolipids is indicative of abnormalities in sphingolipid metabolism which contribute to treatment resistance and prostate cancer progression [7]. Inhibition of the ceramide-S1P signalling axis was able to suppress cancer growth [7, 8]. Thus, the clinical outcomes of men with a poor prognostic lipid profile composed of sphingolipids may be improved by sphingolipid-targeting therapies.

However, in order to integrate selection of patients with a poor prognostic lipid profile into clinical trials, these men must be identifiable via a clinically accessible, regulatory-compliant assay. The 3LS is measured using a high-throughput LC-MS method, which lacks standardisation and validation via regulatory or industry standards [11, 12]. Reproducibility of LC-MS methods between laboratories remains challenging [13] and development of an assay according to National Pathology Accreditation Advisory Council (NPAAC) guidelines is required for clinical implementation [12].

This study aims to develop an accurate prognostic plasma lipid biomarker assay in accordance with the NPAAC guideline [12], that can identify men with mCRPC who have poor OS. Our study consisted of two parts—firstly, optimising a single LC-MS assay capable of quantifying a panel of candidate lipids and ensuring that the assay is accurate, precise, robust and regulatory-compliant [11]. Secondly, running the assay on plasma samples from two cohorts of patients with mCRPC, to develop and validate a risk-score model from the optimum combination of lipids capable of identifying men with poor prognosis.

## Materials and methods

### Patient cohorts

Plasma samples were obtained from two cohorts of men with mCRPC (Discovery & Validation). Samples were collected prior to starting conventional therapy (S1) [4, 6]. The Discovery cohort comprised 105 men with mCRPC commencing taxanes (2006–2015). The Validation cohort comprised 183 men with mCRPC commencing taxanes or ARSIs (2016–2020).

Participants provided written informed consent (Monash Health Institutional Review Board (15571X), Royal Prince Alfred Hospital Human Research Ethics Committee (X14-0406, X19-0320), Australia-New Zealand Clinical Trials Registry ACTRN12607000077460, ACTRN12611000540910).

### Quantitation of plasma lipids

Plasma from the Discovery and Validation Cohorts were analysed using three methods. *Method 1:* Candidate lipids were measured in plasma using a targeted LC-MS assay, developed with quantitation based on calibration standards of reference plasma and adjustment with stable isotope internal standards (S2). *Method 2:* Total cholesterol, high-density lipoprotein (HDL) and triglycerides were measured by enzymatic colorimetric assays using the COBAS 8000 analyser (module C702) (Roche). *Method 3:* High-throughput lipidomic analysis of >300 lipids with relative quantitation was performed using LC-MS as described previously (S3) [4, 6]. Results from the high-throughput lipidomic analysis were used to identify samples with the 3LS.

### Statistical analysis

Statistical analysis was performed with the software R v4.1.1 (referenced in S4) and IBM SPSS v27. OS was calculated from the date of treatment commencement to death and censored at date of last follow-up if death had not occurred.

Pearson’s correlation coefficients (Pearson’s *R*) were used to assess the linear relationship between pairs of lipids (R package ‘ggplot2’ v3.3.5). Univariable Cox regression was used to determine the relationship between lipids and OS (R package ‘survival’ v3.2-13).

Various Cox regression models of OS were built using the Discovery cohort. Least absolute shrinkage and selection operator (LASSO) was used to select predictor variables from different combinations of lipids (R packages ‘survival’ v3.2-13, ‘glmnet’ v4.1-2) [14, 15]. Unnecessary covariates were determined with the minimum value of lambda. The model with the highest concordance (C-statistic) was considered the best model.

The sum of the variables of the Cox regression formula (i.e., the lipid concentrations [*X*] multiplied by the natural logarithm of the hazard ratio (HR) (i.e. the coefficient, [*β*]) of each variable - *β*_*1*_*X*_*1*_ + *β*_*2*_*X*_*2*_ + *…* + *β*_*n*_*X*_*n*_) was used as the risk score. A high score indicates poor prognosis. To determine the optimal cut-point of the score that designates if a person has a good or poor prognosis, the risk score was calculated for each person in the Discovery cohort. The range of scores from the median to the 70^th^ percentile were selected as candidate cut-points for evaluation. The 70^th^ percentile was chosen as the maximum score for evaluation because the proportion of men in the Discovery cohort who were designated as poor prognosis by the 3LS was 30%. The clinical outcomes of the risk groups produced by these candidate cut-points were evaluated using the C-statistic (Cox regression) and the log-likelihood (Weibull regression) (R package ‘survival’ v3.2-13) (i.e., men in the total cohort were split into those with scores above and below each cut-point, and survival outcomes were compared between the two groups). The cut-point that gave the highest C-statistic and log-likelihood was chosen (optimal points coincided). Model performance was assessed within the Validation cohort.

The proportional hazards assumption was checked by residual analysis for variables in the final model (R package ‘survival’ v3.2-13, ‘survminer’ v0.4.9).

Receiver Operating Characteristic (ROC) area under the curve (AUC) was used to assess the model’s ability to predict the 3LS (SPSS).

## Results

### Targeted LC-MS assay development

Our previous lipidomic profiling studies have identified other prognostic ceramides that could enhance the performance of the 3LS [5, 7]. Variations in LC-MS platforms and methodology can influence lipid quantitation, where a biomarker model may perform differently under an alternative platform or methodology. Therefore, in addition to the lipids of the 3LS, we included other ceramides as candidates and developed a targeted LC-MS assay to measure them simultaneously—Cer(d18:1/18:0), Cer(d18:1/22:0), Cer(d18:1/24:0), Cer(d18:1/24:1), Cer(d20:1/24:0), Cer(d20:1/24:1) and PC(16:0/16:0)(S2). SM(d18:2/16:0) was not available for purchase either as a standard (for method development and calibration curves) or as a stable isotope internal standard. We trialled an alternate isoform (SM(d18:1/16:0)) but encountered over-saturation of the LC-MS and problems with reliability during assay development (data not shown). SM(d18:1/16:0) was removed from subsequent analysis. Ceramides are closely associated with risk factors of atherosclerosis such as cholesterol and triglycerides, and these factors may influence assay performance [16]. Thus total cholesterol, HDL and triglycerides were also selected as candidates, and their plasma levels were measured separately by established clinical assays.

The LC-MS assay was developed according to NPAAC guidelines (S2), with linearity for all candidate lipids (mean coefficients of determination 0.973–0.998 (Table S2.4.1)). Inter-assay and intra-assay variability was low, with percentage coefficient of variation <10% for all lipids except Cer(d20:1/24:0) and Cer(d20:1/24:1)(<13%), reflecting their low endogenous concentration (S2.6).

### Study cohorts

**Fig. 1:**
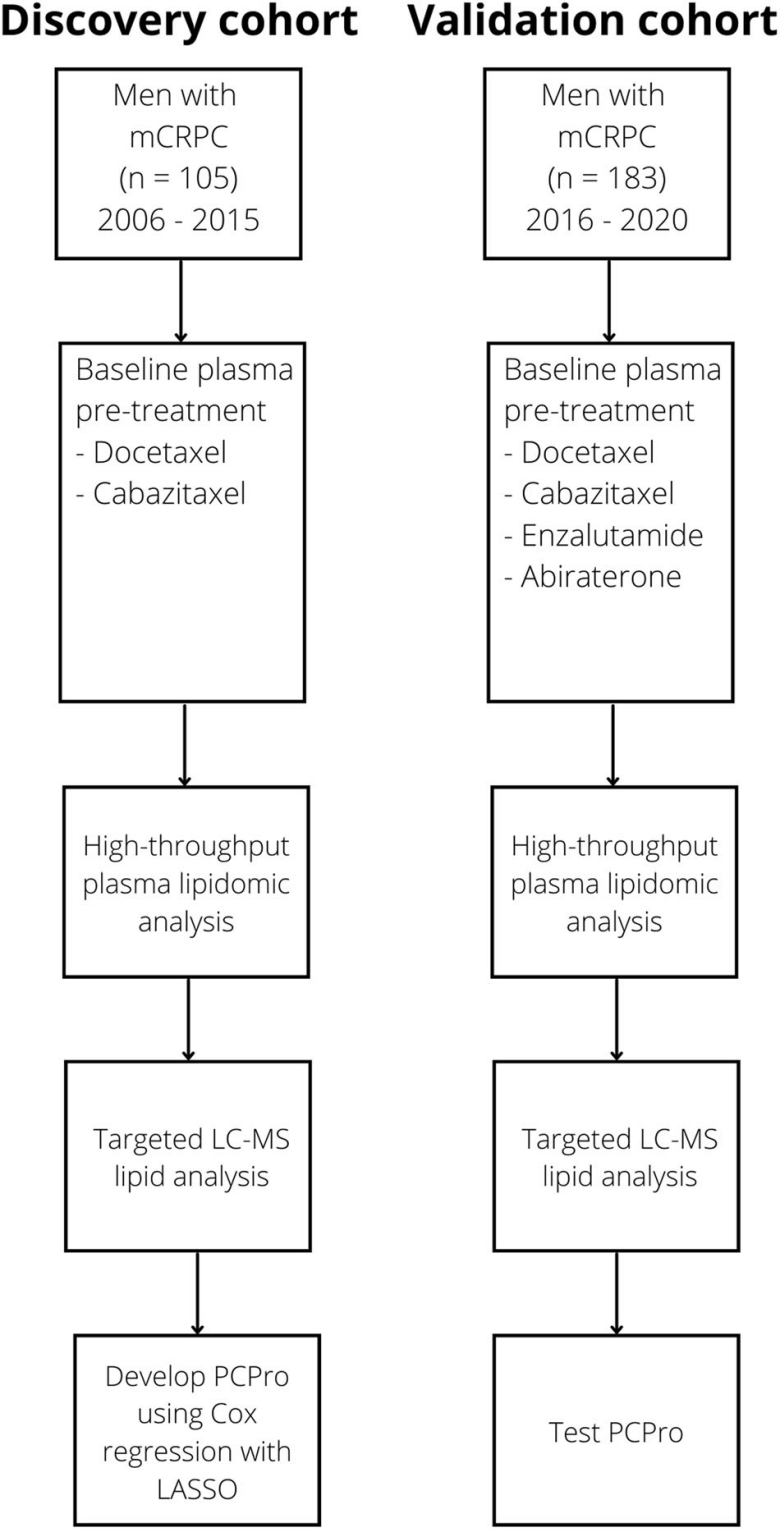
Patient cohorts and analysis strategy. mCRPC metastatic castration-resistant prostate cancer, LC-MS Liquid Chromatography-Mass Spectrometry, LASSO least absolute shrinkage and selection operator.

The characteristics of the Discovery (105 men) and Validation (183 men) cohorts are summarised in Fig. 1, Table S2.2.1. Plasma from the Discovery cohort were obtained at baseline of taxane treatment, which was first line treatment for almost all participants. In contrast, plasma from the Validation cohort were obtained at baseline of ARSI for 70% of the men and 71% of them were first line.

### Comparison between targeted and high-throughput assays

None of the lipids display collinearity with each other, except for Cer(d18:1/24:0) and Cer(d18:1/22:0) with Pearson’s *R* = 0.76 (S5), thus both lipids were never together in the same model during model development.

When the Discovery cohort plasma samples were analysed on the targeted assay, three lipids were associated with OS (*p* < 0.05): Cer(d18:1/24:0), Cer(d18:1/24:1) and Cer(d20:1/24:1). Similar associations were observed when the plasma were analysed on the high-throughput assay, where Cer(d18:1/24:0), Cer(d18:1/24:1), total cholesterol and triglycerides were associated with OS (*p* < 0.05) (Table 1).

**Table 1:** Univariable Cox regression of log2 transformed lipid species concentrations as measured on the targeted and high-throughput assays, in the Discovery and Validation cohorts.

Discovery cohort				
Lipid	High-throughput lipidomic assay		Targeted LC-MS assay	
	Hazard ratio [95% CI]	*p*-value	Hazard ratio [95% CI]	*p*-value
Cer(d18:1/18:0)	1.44 [1.00–2.07]	0.051	1.07 [0.96–1.20]	0.2
Cer(d18:1/22:0)	0.88 [0.52–1.46]	0.6	0.88 [0.48–1.60]	0.7
Cer(d18:1/24:0)	0.60 [0.42–0.87]	**0.007**	0.50 [0.29–0.86]	**0.013**
Cer(d18:1/24:1)	1.92 [1.10–3.32]	**0.021**	2.17 [1.18–4.02]	**0.013**
Cer(d20:1/24:0)			0.85 [0.55–1.31]	0.5
Cer(d20:1/24:1)			1.47 [1.04–2.09]	**0.030**
PC(16:0/16:0)	1.43 [0.76–2.67]	0.3	1.32 [0.69–2.50]	0.4
Total cholesterol	0.52 [0.31–0.87]	**0.014**	0.58 [0.29–1.15]	0.12
HDL			0.88 [0.55–1.43]	0.6
Triglycerides	0.61 [0.43–0.86]	**0.005**	0.68 [0.45–1.02]	0.062
VALIDATION COHORT				
Cer(d18:1/18:0)	1.57 [1.10–2.22]	**0.012**	1.36 [1.02–1.82]	**0.037**
Cer(d18:1/22:0)	0.97 [0.66–1.44]	0.9	1.08 [0.87–1.33]	0.5
Cer(d18:1/24:0)	0.82 [0.55–1.21]	0.3	0.78 [0.62–0.98]	**0.032**
Cer(d18:1/24:1)	1.62 [1.03–2.53]	**0.036**	1.46 [0.92–2.32]	0.11
Cer(d20:1/24:0)	0.95 [0.63–1.43]	0.8	0.96 [0.71–1.31]	0.8
Cer(d20:1/24:1)	1.50 [1.07–2.10]	**0.018**	1.46 [1.06–2.01]	**0.019**
PC(16:0/16:0)	0.95 [0.59–1.53]	0.8	1.04 [0.59–1.85]	0.9
Total cholesterol	0.54 [0.30–0.98]	**0.041**	0.56 [0.34–0.94]	**0.027**
HDL			0.58 (0.39–0.86)	**0.006**
Triglycerides	0.93 [0.73–1.18]	0.6	0.95 (0.72–1.25)	0.7

Bold values indicate statistical significance *p* < 0.05.

Blank cells indicate lipids which were not measured on the high-throughput lipidomic assay.

*Cer* ceramide, *PC* phosphatidylcholine, *HD* high-density lipoprotein, *CI* confidence interval.

Within the Validation cohort, five lipids were associated with OS (*p* < 0.05): Cer(d18:1/18:0), Cer(d18:1/24:0), Cer(d20:1/24:1), total cholesterol and HDL. Similar associations were observed when measured on the high-throughput assay, where Cer(d18:1/18:0), Cer(d18:1/24:1), Cer(d20:1/24:1) and total cholesterol were associated with OS (Table 1).

### Model development (Discovery cohort)

Twelve models consisting of different combinations of candidate lipids were derived (Table 2, S6). Ceramide ratios were included to account for noise reduction and LC-MS variance. Furthermore, ceramide ratios were reported as robust indicators of cardiovascular risk and are less dependent on clinical characteristics [17]. Models 5 and 6 had the highest C-statistic (0.660) and were selected for further evaluation. The optimal cut-points of the risk scores that defines if a person has good or poor prognosis for models 5 and 6 were −1.1903 and −0.817 respectively (S7).

**Table 2:** Details of each of the prognostic models investigated.

Variables entered into the Model^a^	Variables in final model following LASSO shrinkage	C-statistic	*p* value of log-rank test
**Model 1:** Cer(d18:1/18:0), Cer(d18:1/22:0), Cer(d18:1/24:0), Cer(d18:1/24:1), Cer(d20:1/24:0), Cer(d20:1/24:1), PC(16:0/16:0), total cholesterol, HDL, triglycerides	Cer(d18:1/24:0), Cer(d18:1/18:0), Total cholesterol, Triglycerides	0.657	<0.001
**Model 2:** Cer(d18:1/18:0), Cer(d18:1/22:0), Cer(d18:1/24:0), Cer(d18:1/24:1), Cer(d20:1/24:0), Cer(d20:1/24:1), PC(16:0/16:0)	Cer(d18:1/24:0), Cer(d18:1/18:0), Cer(d20:1/24:1)	0.638	<0.001
**Model 3:** Cer(d18:1/18:0), Cer(d18:1/22:0), Cer(d18:1/24:1), Cer(d20:1/24:0), Cer(d20:1/24:1), PC(16:0/16:0), total cholesterol, HDL, triglycerides	Cer(d18:1/24:1), Cer(d18:1/22:0), Cer(d18:1/18:0), Total cholesterol, Triglycerides	0.658	<0.001
**Model 4:** Total cholesterol, HDL, triglycerides	Total cholesterol, HDL, Triglycerides	0.584	0.2
**Model 5:** Cer(d18:1/18:0), Cer(d18:1/22:0), Cer(d18:1/24:0), Cer(d18:1/24:1), Cer(d20:1/24:0), Cer(d20:1/24:1), PC(16:0/16:0), total cholesterol, HDL, triglycerides, ratio of Cer(d18:1/24:0)/Cer(d18:1/24:1)	Cer(d18:1/18:0), Total cholesterol, Triglycerides, Cer(d18:1/24:0)/ Cer(d18:1/24:1)	0.660	<0.001
**Model 6:** Cer(d18:1/18:0), Cer(d18:1/22:0), Cer(d18:1/24:0), Cer(d18:1/24:1), Cer(d20:1/24:0), Cer(d20:1/24:1), PC(16:0/16:0), total cholesterol, HDL, triglycerides, difference between Cer(d18:1/24:0) & Cer(d18:1/24:1)	Cer(d18:1/18:0), Total cholesterol, Triglycerides, Cer(d18:1/24:0) – Cer(d18:1/24:1)	0.660	<0.001
**Model 7:** Cer(d18:1/18:0), Cer(d18:1/22:0), Cer(d18:1/24:0), Cer(d18:1/24:1), Cer(d20:1/24:0), Cer(d20:1/24:1), PC(16:0/16:0), total cholesterol, HDL, triglycerides, ratio of Cer(d18:1/24:0)/Cer(d18:1/24:1), difference between Cer(d18:1/24:0) & Cer(d18:1/24:1)	Cer(d18:1/18:0), Total cholesterol, Triglycerides, Cer(d18:1/24:0)/ Cer(d18:1/24:1)	Model created identical to Model 5	
**Model 8:** Cer(d18:1/18:0), Cer(d18:1/22:0), Cer(d18:1/24:0), Cer(d18:1/24:1), Cer(d20:1/24:0), Cer(d20:1/24:1), PC(16:0/16:0), total cholesterol, HDL, ratio of Cer(d18:1/24:0)/Cer(d18:1/24:1)	Cer(d18:1/18:0), Total cholesterol, Cer(d18:1/24:0)/ Cer(d18:1/24:1)	0.653	<0.001
**Model 9:** Cer(d18:1/18:0), Cer(d18:1/22:0), Cer(d18:1/24:0), Cer(d18:1/24:1), Cer(d20:1/24:0), Cer(d20:1/24:1), PC(16:0/16:0), total cholesterol, HDL, triglycerides, ratio of Cer(d18:1/18:0)/total ceramide, ratio of Cer(d18:1/22:0)/total ceramide^b^, ratio of Cer(d18:1/24:0)/total ceramide^b^, ratio of Cer(d18:1/24:1)/total ceramide^b^, ratio of Cer(d20:1/24:0)/total ceramide^b^, ratio of Cer(d20:1/24:1)/total ceramide^b^	Total cholesterol, Cer(d18:1/18:0)/ Total ceramide, Cer(d18:1/24:0)/ Total ceramide	0.653	<0.001
**Model 10:** Cer(d18:1/18:0), Cer(d18:1/22:0), Cer(d18:1/24:0), Cer(d18:1/24:1), Cer(d20:1/24:0), Cer(d20:1/24:1), PC(16:0/16:0), total cholesterol, HDL, triglycerides, ratio of Cer(d18:1/18:0)/total ceramide, ratio of Cer(d18:1/22:0)/total ceramide^b^, ratio of Cer(d18:1/24:0)/total ceramide^b^, ratio of Cer(d18:1/24:1)/total ceramide^b^, ratio of Cer(d20:1/24:0)/total ceramide^b^, ratio of Cer(d20:1/24:1)/total ceramide^b^, ratio of Cer(d18:1/24:0)/Cer(d18:1/24:1), difference between Cer(d18:1/24:0) & Cer(d18:1/24:1)	Cer(d18:1/18:0)/ Total ceramide, Cer(d18:1/24:0)/ Cer(d18:1/24:1)	0.648	<0.001
**Model 11:** Cer(d18:1/18:0), Cer(d18:1/22:0), Cer(d18:1/24:0), Cer(d18:1/24:1), Cer(d20:1/24:0), Cer(d20:1/24:1), PC(16:0/16:0), total cholesterol, HDL, triglycerides, ratio of Cer(d18:1/18:0)/total cholesterol, ratio of Cer(d18:1/22:0)/total cholesterol, ratio of Cer(d18:1/24:0)/total cholesterol, ratio of Cer(d18:1/24:1)/total cholesterol, ratio of Cer(d20:1/24:0)/total cholesterol, ratio of Cer(d20:1/24:1)/total cholesterol, ratio of PC(16:0/16:0)/total cholesterol	Cer(d18:1/18:0)/ Total cholesterol, Cer(d18:1/24:1)/ Total cholesterol, PC(16:0/16:0)/ Total cholesterol	0.656	<0.001
**Model 12:** Cer(d18:1/18:0), Cer(d18:1/22:0), Cer(d18:1/24:0), Cer(d18:1/24:1), Cer(d20:1/24:0), Cer(d20:1/24:1), PC(16:0/16:0), total cholesterol, HDL, triglycerides, ratio of Cer(d18:1/18:0)/total ceramide^b^, ratio of Cer(d18:1/22:0)/total ceramide^b^, ratio of Cer(d18:1/24:0)/total ceramide^b^, ratio of Cer(d18:1/24:1)/total ceramide^b^, ratio of Cer(d20:1/24:0)/total ceramide^b^, ratio of Cer(d20:1/24:1)/total ceramide^b^, ratio of Cer(d18:1/18:0)/total cholesterol, ratio of Cer(d18:1/22:0)/total cholesterol, ratio of Cer(d18:1/24:0)/total cholesterol, ratio of Cer(d18:1/24:1)/total cholesterol, ratio of Cer(d20:1/24:0)/total cholesterol, ratio of Cer(d20:1/24:1)/total cholesterol, ratio of PC(16:0/16:0)/total cholesterol, ratio of Cer(d18:1/24:0)/Cer(d18:1/24:1), difference between Cer(d18:1/24:0) & Cer(d18:1/24:1)	Total cholesterol, Cer(d18:1/18:0)/ Total ceramide, Cer(d18:1/18:0)/ Total cholesterol, PC(16:0/16:0)/ Total cholesterol, Cer(d18:1/24:0)/ Cer(d18:1/24:1)	0.649	<0.001

*HR* hazard ratio, *C-statistic* concordance, *Cer* Ceramide, *PC* phosphatidylcholine, *HDL* high-density lipoprotein.

^a^The values of the lipids measured by LC-MS are in mg/L. The values of lipids measured by enzymatic colorimetric assays are in mmol/L.

^b^Total ceramide = Cer(d18:1/18:0) + Cer(d18:1/22:0) + Cer(d18:1/24:0) + Cer(d18:1/24:1) + Cer(d20:1/24:0) + Cer(d20:1/24:1).

Patients classified as poor prognosis by either model had significantly shorter OS than patients classified as good prognosis (model 5: median OS 12.0 months vs. 24.2 months, HR 3.75 [95% confidence interval (CI) 2.29–6.15], *p* < 0.001; model 6: median OS 12.2 months vs. 26.4 months, HR 3.62 [2.21–5.94], *p* < 0.001). The HR of Model 5 was higher than Model 6, therefore, Model 5 was chosen as the optimal model and designated as “PCPro”. The Cox proportional hazards assumptions for PCPro were verified by residuals analysis (S8).

### PCPro performance within the Validation Cohort

**Fig. 2:**
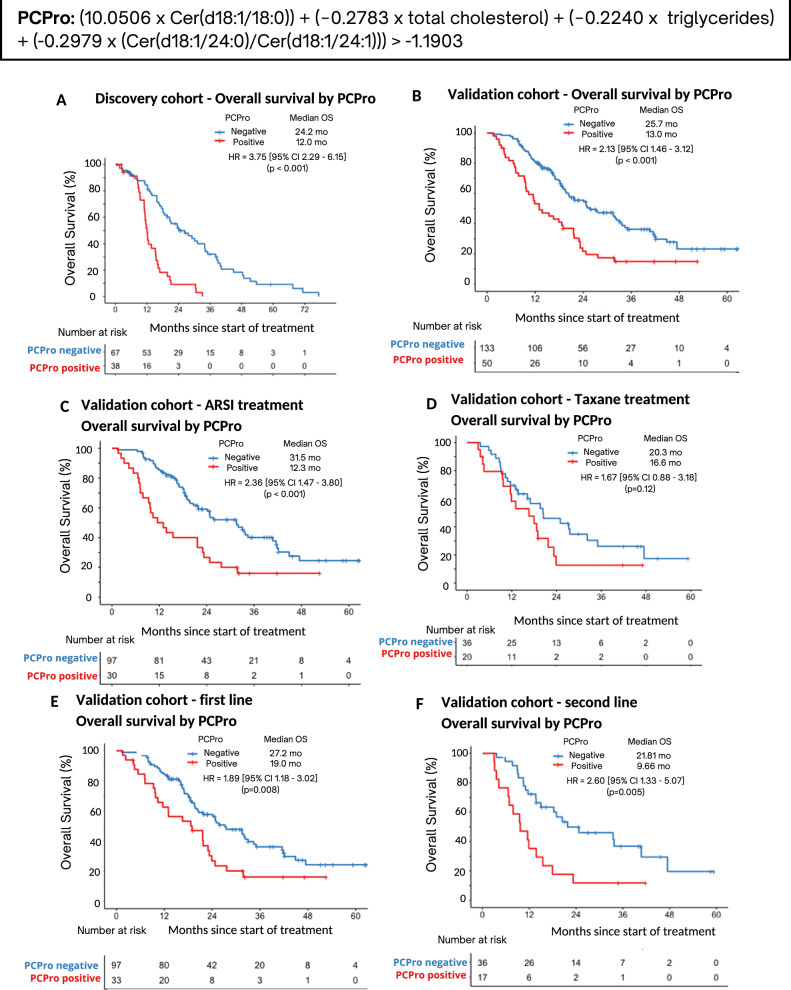
Kaplan-Meier survival analysis of overall survival by PCPro in the Discovery and Validation cohorts. **A** Survival in the Discovery cohort, **B** Survival in the Validation cohort, **C** Survival in those treated with ARSI in the Validation cohort, **D** Survival in those treated with taxane chemotherapy in the Validation cohort, **E** Survival in those treated with first line treatment in the Validation cohort, and **F** Survival in those treated with second line treatment in the Validation cohort. ARSI androgen receptor signalling inhibitor, HR hazard ratio, mo months, OS overall survival.

PCPro classified 50 of 183 men (27%) as poor prognostic (PCPro-positive) in the Validation cohort. Median OS of the PCPro-positive group was significantly shorter than the PCPro-negative group (13.0 vs 25.7 months, HR = 2.13 [95% CI 1.46–3.12], *p* < 0.001) (Fig. 2).

Sub-group analysis by therapy showed that for those treated with an ARSI, median OS was significantly shorter for PCPro-positive men compared to PCPro-negative men (12.3 vs 31.5 months, HR = 2.36 [95% CI 1.47–3.80], *p* < 0.001). Although OS was shorter amongst those treated with taxanes who were PCPro positive compared to PCPro negative, it was not statistically significant (median OS 16.6 vs 20.3 months, HR = 1.67 [95% CI 0.88–3.18], *p* = 0.12).

Sub-group analysis by treatment line showed that for men on first or second-line therapy, those who were PCPro-positive had shorter OS than men who were PCPro-negative (first-line: median OS 19.0 vs 27.2 months, HR = 1.89 [95% CI 1.18–3.02], *p* = 0.008; second-line: median OS 9.7 vs 21.8 months, HR = 2.60 [95% CI 1.33–5.07], *p* = 0.005).

Analyses of progression-free survival (PFS) by cohort, therapy or treatment line showed that men who were PCPro-positive had shorter PSA-PFS or radiographic-PFS compared to men who were PCPro-negative (*p* < 0.05, S9).

### Comparison of model to 3LS and clinicopathological factors

A high percentage of PCPro-positive patients have the 3LS (Discovery cohort: 68%; Validation cohort: 70%). The ROC AUC of the ability of PCPro to predict 3LS was 0.869 for the Discovery cohort and 0.751 for the Validation cohort (S10).

When PCPro was modelled with clinicopathological factors in multivariable Cox regression, PCPro was an independent predictor of OS (*p* < 0.05) in both the Discovery and Validation cohorts. Alkaline phosphatase was an additional independent predictor in the Discovery cohort and haemoglobin and albumin were additional independent predictors in the Validation cohort (S11). In bivariable Cox regression analysis, PCPro was an independent predictor of OS whereas diabetes status was not. PCPro was not associated with the presence of diabetes (S11.3). Other cardiovascular risk factors including age, weight, BMI and statin-use were not associated with OS (S11).

## Discussion

In summary, we have developed and validated PCPro, a novel lipid-based risk-score associated with poor prognostic mCRPC. PCPro effectively identified men with shorter OS in two independent cohorts. The prognostic ability of PCPro remained evident when cohorts were stratified by therapy or treatment line. PCPro can be performed in hospital laboratories and may be used to identify men with mCRPC for prospective studies of metabolic therapy.

The National Cancer Institute’s strategy for biomarker discovery demands that following assay development and analytical validation, a biomarker must progress through a clinical validation pathway through to commercialisation and regulatory approval—a process that only a fraction achieve [18]. Although biomarker research is academically interesting [19], for biomarkers to be clinically useful, they must enact clinical decisions that improve patient outcomes [20]. Development of a biomarker using an NPAAC-concordant assay provides a pipeline towards clinically meaningful implementation.

An advantage of PCPro over high-throughput LC-MS is the use of unique stable isotope-labelled internal standards for each lipid. Internal standards account for recovery variance and matrix effects [13]. Ideally, an internal standard should be used for each species analysed by LC-MS, however this is often prohibited by cost and availability when research assays include high lipid numbers [21, 22]. Internal standards were used for each analyte measured in PCPro, whereas in the high-throughput assay only a single internal standard was used for the entire ceramide class [23]. A further advantage is that PCPro includes variables that are inversely correlated with survival (i.e. higher Cer(d18:1/24:0), total cholesterol and triglycerides are associated with longer OS, whereas higher Cer(d18:1/18:0) and Cer(d18:1/24:1) are associated with shorter OS). The inclusion of these variables may be related to their biology and adjusts for confounding effects, as ceramide, cholesterol and triglyceride synthesis and metabolism are co-regulated [24–26].

Our previous work showed that men with mCRPC with alterations in their lipidomic profile have poor OS [4–7]. The alteration in the lipidome extends well beyond the lipids included in PCPro. It would be impractical to include all these lipids, and rather, PCPro acts as an indicator for underlying metabolic changes.

The precision-oncology era is characterised by personalised therapeutics, and the development of specific biomarkers are crucial to delivering treatment to those who benefit most, sparing non-responders the cost and side-effects of treatment [27]. Our next step is to integrate PCPro into clinical trials, to select patients to receive ceramide-targeting therapies in addition to standard care. Potential ceramide-targeting therapies are sphingosine kinase (SPHK) inhibitors, which inhibit the conversion of ceramide into S1P. SPHK inhibitors display anti-cancer effects in vitro and in mouse models, including prostate cancer models [8, 28, 29]. Our previous work showed that SPHK inhibitors overcome enzalutamide resistance in prostate cancer cell lines and explants [7]. Statins are able to decrease circulating levels of ceramides; however, retrospective studies of statins in addition to standard care for prostate cancer showed mixed results in improvement of clinical outcomes [30–32]. Perhaps treating patients who are PCPro-positive with metabolic therapies will enrich for response. We hypothesise that PCPro has potential as a predictive biomarker, not just a prognostic tool, and this will be evaluated in prospective clinical trials.

Strengths of this study include the rigorous NPAAC framework in which the signature was developed, and that the analysis of independent cohorts delivered similar observations. A study limitation was the heterogeneity between the Discovery and Validation cohorts. The Discovery cohort was all docetaxel-treated, while the Validation had only a subset treated with taxanes and not all were first-line treatment. Consequently, the shorter OS in PCPro-positive men was not statistically significant, due to smaller numbers. However, PFS was significantly shorter amongst men treated with taxanes in the Validation cohort who were PCPro-positive compared to PCPro-negative, strengthening the support for the discriminative ability of PCPro (S9). This was also reassuring as the results support PCPro’s prognostic utility across a range of treatments, not just a single modality. Another study limitation is the lack of ethnic diversity. Over 90% of participants were Caucasian, recruited from an Australian population where there is little African ancestry. The range of treatments used is similar to other developed countries, however the signature should be validated in an ethnically diverse population.

## Conclusions

We have developed PCPro, a novel lipid biomarker capable of prospectively identifying men with mCRPC with shorter OS. We hypothesise that this poor prognostic circulating lipid profile is metabolically actionable though drug and lifestyle interventions. PCPro will allow us to identify men for prospective clinical trials of agents targeting lipid metabolism.

### Supplementary information

Data supplement

## Data Availability

Method development and validation data are included in the data supplement. Individual lipid results are available upon request. Clinical information of individual patients cannot be provided due to the ethics restrictions.
