# Supplementary material for: PCPro: a clinically accessible, circulating lipid biomarker signature for poor-prognosis metastatic prostate cancer
Source: Prostate Cancer Prostatic Dis. 2023 May 5;27(1):136–43. doi: 10.1038/s41391-023-00666-2 (PMC10876475; doi:10.1038/s41391-023-00666-2)
Supplement: Supplementary file 1 — Data supplement [file 41391_2023_666_MOESM1_ESM.docx]

# DATA SUPPLEMENT - PCPro: a clinically accessible, circulating lipid biomarker signature for poor-prognosis metastatic prostate cancer

[DATA SUPPLEMENT - PCPro: a clinically accessible, circulating lipid biomarker signature for poor-prognosis metastatic prostate cancer 1](#_Toc128665081)

[S1. Patient cohorts 3](#_Toc128665082)

[S1.1 Blood collection 3](#_Toc128665083)

[S1.1.1 Discovery cohort 3](#_Toc128665084)

[S1.2.2 Validation cohort 3](#_Toc128665085)

[S1.2 Patient characteristics 4](#_Toc128665086)

[S2. Targeted assay method development and validation 5](#_Toc128665087)

[S2.1. Chemicals and Reagents 5](#_Toc128665088)

[S2.1.1 Synthesis of ceramide(d20:1/24:0) and ceramide(d20:1/24:1) 5](#_Toc128665089)

[S2.1.1.1 Ceramide(d20:1/24:0) synthesis 5](#_Toc128665090)

[S2.1.1.2 Ceramide(d20:1/24:1) synthesis 5](#_Toc128665091)

[S2.2 LC-MS assay development methods 6](#_Toc128665092)

[S2.2.1 Preparation of Stock and Working Solutions 6](#_Toc128665093)

[S2.2.2 Lipid extraction of plasma samples 6](#_Toc128665094)

[S2.2.3 LC-MS analysis 6](#_Toc128665095)

[S2.2.4 Determination of plasma lipid concentration 6](#_Toc128665096)

[S2.2.5 Quality Controls 7](#_Toc128665097)

[S2.2.6 Recovery of lipids and matrix effects 7](#_Toc128665098)

[S2.2.7 Analysis of clinical plasma samples 7](#_Toc128665099)

[S2.2.8 Optimal LC-MS conditions 7](#_Toc128665100)

[S2.3 M/z MRM pairs and internal standards 8](#_Toc128665101)

[S2.4 Linearity of calibration curves, analyte recovery and matrix effects 10](#_Toc128665102)

[S2.5 Quality control (QC) samples and stability assessments 11](#_Toc128665103)

[S2.6 Inter- and Intra- assay variation 13](#_Toc128665104)

[S2.7 Limits of quantification 15](#_Toc128665105)

[S2.8 Ion suppression assessment 16](#_Toc128665106)

[S2.9 Endogenous lipid concentrations in FFP-1 19](#_Toc128665107)

[S3. High-throughput plasma lipidomic analysis 20](#_Toc128665108)

[S3.1 High-throughput plasma lipidomic analysis method 20](#_Toc128665109)

[S3.2. Calculation of the three-lipid signature (3LS) 20](#_Toc128665110)

[S4. References for statistical analysis methods 21](#_Toc128665111)

[S4.1 R statistical software environment v4.1.1 21](#_Toc128665112)

[S4.2 IBM SPSS v27 21](#_Toc128665113)

[S5. Correlation matrix of lipid concentrations measured by the targeted assay 22](#_Toc128665114)

[S6. Models developed by Cox Regression with the LASSO shrinkage method 24](#_Toc128665115)

[S7. Optimal cut-point determination 29](#_Toc128665116)

[S7.1 Cut-points tested for Model 5 29](#_Toc128665117)

[S7.2 Cut-points tested for Model 6 30](#_Toc128665118)

[S8. Verification of Cox proportional hazards assumptions for PCPro by Residuals testing in the Discovery Cohort 31](#_Toc128665119)

[S8.1 Schoenfeld residuals 31](#_Toc128665120)

[S8.2 Martingale residuals 32](#_Toc128665121)

[S9. Kaplan-Meier Survival analysis of progression free survival (PFS) by PCPro in the Discovery and Validation cohorts 33](#_Toc128665122)

[S10. Correlation of PCPro with the three-lipid prognostic signature 34](#_Toc128665123)

[S10.1 Correlation in the Discovery cohort 34](#_Toc128665124)

[S10.2 Correlation in the Validation cohort 35](#_Toc128665125)

[S11. Comparison of PCPro to clinicopathological factors 36](#_Toc128665126)

[S11.1 Cox Regression Analysis with clinicopathological factors 36](#_Toc128665127)

[Discovery cohort 36](#_Toc128665128)

[Validation cohort 36](#_Toc128665129)

[S11.2 Comparison of PCPro to Halabi’s nomogram and a clinicopathological model 37](#_Toc128665130)

[Discovery cohort 37](#_Toc128665131)

[Validation cohort 38](#_Toc128665132)

[S11.3 Relationship between PCPro and diabetic status in the Discovery cohort 39](#_Toc128665133)

[S11.4 Univariable and bivariable Cox Regression of age and PCPro 39](#_Toc128665134)

[S11.4.1 Univariable and bivariable Cox Regression of age and PCPro in the Discovery Cohort 39](#_Toc128665135)

[S11.4.2 Univariable and bivariable Cox Regression of age and PCPro in the Validation Cohort 39](#_Toc128665136)

[S11.5 Univariable Cox regression of traditional cardiovascular risk factors and PCPro 39](#_Toc128665137)

[S11.6 Relationship between PCPro and statin use in the Discovery cohort 40](#_Toc128665138)

[Reference List 41](#_Toc128665139)

# S1. Patient cohorts

These cohorts are subsets of cohorts in previous lipidomic studies from which there were remaining plasma aliquots – the Discovery cohort is a subset of Phase 1 and 2 cohorts in Lin *et al* 2017,[1] whereas the Validation cohort is a subset of the cohort in Lin et al 2021.[2]

## S1.1 Blood collection

### S1.1.1 Discovery cohort

Peripheral blood samples were collected in BD Vacutainer tubes containing K_2_EDTA, centrifuged at 3000g for 5 minutes at room temperature, and the plasma aliquoted into cryovials and stored at -80°C cryostorage until required.

### S1.2.2 Validation cohort

Whole blood was collected into 10mL EDTA-containing tubes and two-step centrifugation performed to separate plasma and buffy coat. Blood was first centrifuged at 1600g for 15 minutes and the supernatant was transferred to a fresh tube, where it was centrifuged again at 5000g for 10 minutes. Aliquots of the plasma after the second centrifugation were stored at -80°C until required.

## S1.2 Patient characteristics

**Table S1.2.1: Patient characteristics of the Discovery and Validation cohorts**

|  | **Discovery cohort** | **Validation cohort** |
| --- | --- | --- |
| **Number of men** | 105 | 183 |
| **Median age, years** [Q1, Q3] | 70 [64, 76] | 73 [66, 80] |
| **Median follow-up time for surviving patients, months** [Q1, Q3] | 12 [4, 25] | 30 [16,46] |
| **Median follow-up time for all patients, months** [Q1, Q3] | 16 [11, 28] | 19 [11, 31] |
| **Median Overall Survival, months** [Q1, Q3] | 18 [12, 34] | 22 [12, 35] |
| **Deaths** | 86 (82%) | 120 (66%) |
| **Baseline Prostate Specific Antigen, ng/mL** [Q1, Q3] | 128 [48, 327] | 31 [12,79] |
| **Gleason grade**  ≤6  7  8  ≥9  Missing | 8 (8%)  22 (21%)  17 (16%)  33 (31%)  25 (24%) | 10 (5%)  35 (19%)  23 (13%)  85 (46%)  30 (16%) |
| **Baseline metastasis**  Bone  Soft tissue  Missing | 92 (88%)  36 (34%)  9 (9%) | 160 (87%)  93 (51%)  0 (0%) |
| **Treatment**  Docetaxel  Cabazitaxel  Enzalutamide  Abiraterone | 104 (99%)  1 (1%)  0  0 | 48 (26%)  8 (4%)  88 (48%)  39 (21%) |
| **Line of treatment**  First line  Second Line  Third Line | 101 (96%)  3 (3%)  1 (1%) | 130 (71%)  53 (29%)  0 (0%) |
| **Subsequent lines of treatment**  None  1 line  2 lines  3 lines  ≥4 lines | 28 (27%)  36 (34%)  25 (24%)  12 (11%)  4 (4%) | 87 (48%)  57 (31%)  31 (17%)  8 (4%)  0 (0%) |
| **Hospital**  Royal Prince Alfred Hospital/Lifehouse  Concord Repatriation General Hospital  Westmead Hospital  Calvary Mater Hospital  St George Hospital  Sydney Adventist Hospital  Coffs Harbour Hospital  Monash Health  Epworth Freemasons  St Vincent’s Hospital | 47 (45%)  19 (18%)  12 (11%)  10 (10%)  3 (3%)  6 (6%)  8 (8%)  0 (0%)  0 (0%)  0 (0%) | 41 (22%)  12 (7%)  0 (0%)  0 (0%)  0 (0%)  4 (2%)  3 (2%)  77 (42%)  17 (9%)  29 (16%) |
| **Three-Lipid signature positive** | 31 (30%) | 69 (38%) |

Abbreviations: Q1, first quartile; Q3, third quartile

# S2. Targeted assay method development and validation

## S2.1. Chemicals and Reagents

Standards and deuterated standards of ceramides and phosphatidylcholine were purchased from Cayman Chemicals (Ann Arbor, MI). Quality control (QC) standards (which requires an independent source) for Cer(d18:1/18:0), Cer(d18:1/22:0), Cer(d18:1/24:0) and Cer(d18:1/24:1) were purchased from Toronto Research Chemicals (TRC) (Toronto, ON), and for PC(16:0/16:0) from Avanti Lipids (Birmingham, AL).

All high-performance liquid chromatography (HPLC) solvents were LC-MS grade. Acetonitrile, isopropanol, methanol, and 1-Butanol were sourced from LiChrosolv, Merck (Darmstadt, Germany). Ammonium formate was sourced from Sigma-Aldrich. Ultrapure water was produced in house using the Puris-esse water purification system (Model: Esse-Up Analy-TOC-M, Rotek Australia, Bayswater, Australia).

Cer(d20:1/24:0) and Cer(d20:1/24:1) were synthesised in-house (S2.1.1).

### S2.1.1 Synthesis of ceramide(d20:1/24:0) and ceramide(d20:1/24:1)

Ceramides were synthesised by amide coupling reaction between long chain acids and sphingosines using hydroxybenzotriazole activated esters. All chemicals for the synthesis of Cer(d20:1/24:0) and Cer(d20:1/24:1) were purchased from Cayman chemicals except for 1-hydroxybenzotriazole hydrate which was purchased from TRC and triethylamine and 1-ethyl-3-(3-dimethylaminopropyl)carbodiimide) which were purchased from Sigma.

### S2.1.1.1 Ceramide(d20:1/24:0) synthesis

A solution of triethylamine (15 μmol) and d(20:1) sphingosine (3.3 mg, 10 μmol) in anhydrous dichloromethane (10 mL) was added dropwise to a solution of lignoceric acid (3.6 mg, 10 μmol), 1-Hydroxybenzotriazole Hydrate (2.0 mg, 15 μmol) and triethylamine (15 μmol) in anhydrous dichloromethane/acetonitrile (9:1) over 2 minutes. The mixture was stirred for 3 hours before being quenched with aqueous NaHCO_3_ (5%, 20 mL). The aqueous layer was discarded, and the organic layer washed with aqueous HCl (0.1 M, 20 mL) and water (20 mL) before being evaporated to dryness. The crude mixture was then redissolved in methanol and purified by HPLC, to yield 1.42 mg of pure ceramide(d20:1/24:0). M/z MRM pairs chosen were 678.6/292.2, and 678.6/280.35 and HPLC retention time was 7.3 minutes.

### S2.1.1.2 Ceramide(d20:1/24:1) synthesis

A solution of triethylamine (15 μmol) and d(20:1) sphingosine (3.3 mg, 10 μmol) in anhydrous dichloromethane (10 mL) was added dropwise to a solution of nervonic acid (3.6 mg, 10 μmol), 1-hydroxybenzotriazole hydrate (2.0 mg, 15 μmol) and triethylamine (15 μmol) in anhydrous dichloromethane/acetonitrile (9:1) over 2 minutes. The mixture was stirred for 3 hours before being quenched with aqueous NaHCO_3_ (5%, 20 mL). The aqueous layer was discarded, and the organic layer washed with aqueous HCl (0.1 M, 20 mL) and water (20 mL) before being evaporated to dryness. The crude mixture was then redissolved in methanol and purified by HPLC, to yield 4.90 mg of pure ceramide(d20:1/24:1). M/z MRM pairs chosen were 676.6/292.35, 676.6/280.3 and 676.6/310.2 and HPLC retention time was 6.7 minutes.

## S2.2 LC-MS assay development methods

### S2.2.1 Preparation of Stock and Working Solutions

Stock solutions of standards (1mg/mL) were prepared in isopropanol. A working solution was prepared by diluting stock solutions with 1-butanol/methanol (v/v 1:1) (BuMe) to concentrations of 0.25mg/L Cer(d18:1/18:0), 3.75mg/L Cer(d18:1/22:0), 6.5mg/L Cer(d18:1/24:0), 5.25mg/L Cer(d18:1/24:1), 0.1mg/L Cer(d20:1/24:0, 0.1mg/L Cer(d20:1/24:1) and 20mg/L PC(16:0/16:0).

A mixed solution of internal standards (IS) was combined with a protein precipitation solution (PPS) consisting of 10mM ammonium formate in BuMe. The concentration of the internal standards in PPS were 0.5mg/L D_7_-Cer(d18:1/18:0), 0.65mg/L D_7_-Cer(d18:1/24:0), 0.52mg/L D_7_-Cer(d18:1/24:1) and 2.5mg/L D_9_-PC(16:0/16:0).

A single source of thawed fresh frozen plasma (FFP) (FFP-1) was obtained from the Royal Prince Alfred Blood Bank. This was used for method optimisation, creation of standard curves and quality control (QC) samples.

### S2.2.2 Lipid extraction of plasma samples

800μL of IS-PPS was combined with 100μL of plasma and 100μL of BuMe, vortexed for 10 seconds, then sonicated for 60 mins in a water bath at 20°C and finally, centrifuged at 16,000g for 10 minutes. 750μL of the supernatant was transferred to a glass vial for LC-MS analysis.

### S2.2.3 LC-MS analysis

Lipid extracts were analysed on a Shimadzu LCMS-8050 using multiple-reaction monitoring (MRM). Optimal mass spectrometer conditions (S2.2.8) for the analytes were determined by infusing individual lipid standards at 1μg/mL.

### S2.2.4 Determination of plasma lipid concentration

The concentration of lipids in clinical plasma samples were normalised to ISs and calculated from calibration standards consisting of spiked FFP-1 that were freshly prepared and included in every analytical run. Spiked FFP-1 were prepared by mixing 100μl of FFP-1 with 100μl of lipid standard and extracted in the same manner as the clinical plasma samples (replacing BuMe and plasma with 200μl of spiked FFP-1, and adding 800μL of IS-PPS, as per S2.2.2). The highest spike concentration (making up the 100μl of lipid standard) was the same as the working solution of the lipid in pure BuMe. Subsequent spike concentrations were respectively 80%, 60%, 40%, 20% and 0% of the working solution. The concentration of the analytes in the calibration standards was pre-determined by the method of standard addition which was performed on eight replicate sets of the calibration standards.[3] For each replicate set, a calibration curve was obtained by simple linear regression of the standards, and the concentration of analytes in blank FFP-1 (blank spike) was extrapolated from the regression line. The mean concentration in blank FFP-1 across the eight replicates was used to calculate the concentration of the analytes at each point on the calibration curve.

### S2.2.5 Quality Controls

QCs, consisting of spiked FFP-1 at three concentrations for each analyte (detailed in S2.5), are included in every analytical run to monitor LC-MS method performance. To minimise error, independent sources of base materials should be used for QCs and calibration standards, thus the analytes for the QCs were sourced from an alternate company to the lipids used in our calibration curves where available.[4] QC concentrations were based on the distribution of concentrations detected in plasma samples in our previously published studies.[1, 5, 6]

### S2.2.6 Recovery of lipids and matrix effects

FFP and aqueous calibration standards were analysed in eight replicate runs for linearity of response of analytes. Recovery of the analytes was calculated as the calibration curve slope in FFP divided by the curve slope in water on the same analytical run. Reduction in recovery of <10% was considered non-significant. Matrix effects were determined by comparing analyte concentrations between spikes (i.e. lipid standards as detailed in S2.2.4) added prior and following lipid extraction of three different thawed FFP samples, with differences of <10% considered non-significant. The highest calibration spike concentration was used. For pre-extraction spikes, the spike was added to the FFP before lipid extraction. For post-extraction spikes, the spike was added after the sonication step of the lipid extraction process, prior to the centrifugation step.

### S2.2.7 Analysis of clinical plasma samples

Discovery cohort samples were extracted and analysed across two runs. Validation cohort samples were extracted and analysed in 7 runs over 6 days. Each run included FFP-1 calibration standards and QCs. All patients from the Discovery and Validation cohorts had previously been assayed for the presence or absence of the poor prognostic 3LS (S2).[1, 6]

### S2.2.8 Optimal LC-MS conditions

Optimal LC-MS conditions were as follows: interface temperature, 300°C; nebulising gas flow, 1.6L/min; drying gas flow, 10L/min; heating gas flow, 10L/min; column oven temperature, 60°C; ion spray voltage, 4.0kV. Chromatographic separation was performed on an ACE Excel 2 C18, 50mm column (Advanced Chromatography Technologies). Mobile phase consisted of (A) 50% water, 30% acetonitrile, 20% isopropanol with 10mM ammonium formate and (B) 1% water, 9% acetonitrile, 90% isopropanol with 10mM ammonium formate. Injection volume was 5μL and the flow rate was set to 0.8mL/min. The gradient was as follows: starting with 10% B, increasing to 45% B over 1.9 minutes, then to 53% over 0.01 minutes, to 65% over 4.39 minutes, to 89% over 0.1 minutes, to 92% over 1.3 minutes and to 100% over 0.1 minutes. The solvent was then held at 100% B for 0.5 minutes. Solvent was decreased from 100% B to 10% B over 0.1 minutes and held for an additional 2.1 minutes (for a total cycle time of 10.5 minutes). Dwell time was set at 24ms for each of the signal transitions except for the transitions belonging to PC(16:0/16:0) and its’ corresponding internal standard, where the dwell times were set at 3ms.

## S2.3 M/z MRM pairs and internal standards

**Table S2.3.1 m/z MRM pairs, internal standards, and retention time for each lipid**

| **Lipid species** | **m/z MRM pair^*^** | **Corresponding deuterated internal standard** | **Retention time (minutes)** |
| --- | --- | --- | --- |
| Cer(d18:1/18:0) | 566.5/264.3  566.5/282.3 | D_7_-Cer(d18:1/18:0) | 4.6 |
| Cer(d18:1/22:0) | 622.6/264.2  622.6/282.2 | D_7_-Cer(d18:1/24:1) | 5.95 |
| Cer(d18:1/24:0) | 650.6/264.3  650.6/282.3 | D_7_-Cer(d18:1/24:0) | 6.7 |
| Cer(d18:1/24:1) | 648.6/264.3  648.6/282.3 | D_7_-Cer(d18:1/24:1) | 5.9 |
| Cer(d20:1/24:0) | 678.6/292.2  678.6/292.2  678.6/280.35 | D_7_-Cer(d18:1/24:0) | 7.3 |
| Cer(d20:1/24:1) | 676.6/292.35  676.6/280.3  676.6/310.2 | D_7_-Cer(d18:1/24:0) | 6.7 |
| PC(16:0/16:0) | 734.5/184.55  734.5/478.6  734.5/496.7 | D_9_-PC(16:0/16:0) | 4.3 |
| D_7_-Cer(d18:1/18:0) | 573.5/259.3  573.5/289.3 |  | 4.6 |
| D_7_-Cer(d18:1/24:0) | 657.6/271.3  657.6/289.3 |  | 6.7 |
| D_7_-Cer(d18:1/24:1) | 655.6/271.3  655.6/289.3 |  | 5.9 |
| D_9_-PC(16:0/16:0) | 744.5/185.2  744.5/488.3  744.5/506.5 |  | 4.3 |

Abbreviations: m/z, mass to charge ratio; MRM, multiple reaction monitoring; Cer, Ceramide; PC, phosphatidylcholine

^*^ For each M/Z MRM pair, the first pair is the quantitating ion and subsequent pairs are qualifying ions

Due to the several-fold higher concentration of PC(16:0/16:0) in endogenous plasma, the m/z MRM pairs for PC(16:0/16:0) were adjusted in order to attenuate the signal. This involved increasing the m/z of the product ions for PC(16:0/16:0) by 0.2 to give the final m/z MRM pairs described above. The optimised collision energies were also adjusted by +10. The corresponding ISs were adjusted accordingly.

No signals of the respective ISs were observed in extracted blank FFP (when extracted without the presence of ISs), showing no interference coming from the IS. The retention time of respective endogenous species matched the ISs. D_7_-cer(d18:1/24:1) was used as the IS for both Cer(d18:1/22:0) and Cer(d18:1/24:1), and D_7_-Cer(d18:1/24:0) was used as the IS for Cer(d18:1/24:0), Cer(d20:1/24:0) and Cer(d20:1/24:1) due to their similar retention times and the unavailability of a deuterated standard for Cer(d18:1/22:0), Cer(d20:1/24:0) and Cer(d20:1/24:1).

The LC-MS approach provided baseline separation of all lipid species (figure S2.3.1). **
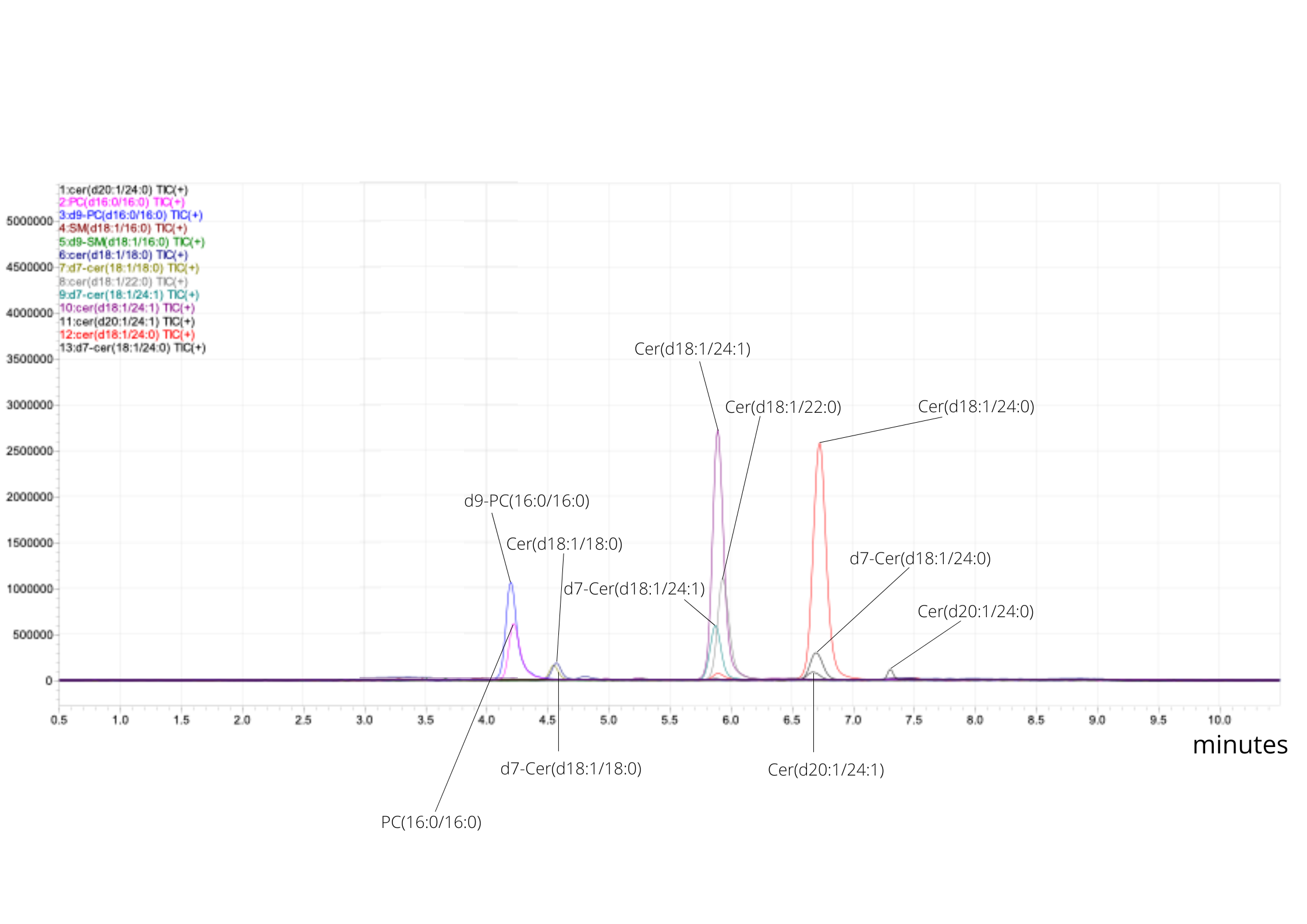
**

**Figure S2.3.1: LC-MS chromatograms of each of the analytes of interest in FFP-1**

Abbreviations: Cer, ceramide; PC, phosphatidylcholine

## S2.4 Linearity of calibration curves, analyte recovery and matrix effects

The calibration curves of lipid standards prepared in plasma showed linearity for all candidate lipids with mean coefficients of determination of 0.973 – 0.998 (Table S2.4.1). The recovery of the spiked lipid standards from plasma ranged from 93–102% for all lipids except for cer(d20:1/24:0) at 72% (Table S2.4.2). In order to include a wide variety of analytes in a single assay, this low recovery value was considered acceptable. Plasma matrix effects were minimal, with pre-extraction peak areas being 97 – 115% of the post extraction peak areas across all lipid species (Table S2.4.2).

**Table S2.4.1: Results of linearity assessment for each lipid in FFP across each run replicate for the determination of the concentrations on the calibration curve**

| **Coefficient of determination (R^2^)** | | | | | | | |
| --- | --- | --- | --- | --- | --- | --- | --- |
| **Run no:** | **Cer (d18:1/18:0)** | **Cer (d18:1/22:0)** | **Cer (d18:1/24:0)** | **Cer (d18:1/24:1)** | **Cer (d20:1/24:0)** | **Cer (d20:1/24:1)** | **PC (16:0/16:0)** |
| **1** | 0.9642 | 0.994 | 0.9918 | 0.9981 | 0.9987 | 0.9984 | 0.9923 |
| **2** | 0.9937 | 0.9979 | 0.9864 | 0.9997 | 0.9879 | 0.9926 | 0.9906 |
| **3** | 0.9937 | 0.9953 | 0.9947 | 0.9891 | 0.995 | 0.9982 | 0.9768 |
| **4** | 0.9856 | 0.9986 | 0.9763 | 0.999 | 0.9897 | 0.994 | 0.9938 |
| **5** | 0.9714 | 0.999 | 0.9962 | 0.9992 | 0.9771 | 0.9965 | 0.993 |
| **6** | 0.9739 | 0.9949 | 0.9875 | 0.9979 | 0.987 | 0.9844 | 0.9457 |
| **7** | 0.9628 | 0.999 | 0.9889 | 0.9989 | 0.9952 | 0.9987 | 0.9344 |
| **8** | 0.9424 | 0.99 | 0.9396 | 0.9982 | 0.9949 | 0.9988 | 0.9922 |
| **Mean** | 0.973 | 0.996 | 0.983 | 0.998 | 0.991 | 0.995 | 0.977 |
| **%CV** | 1.68 | 0.30 | 1.76 | 0.32 | 0.65 | 0.46 | 2.28 |

Abbreviations: Cer, Ceramide; PC, phosphatidylcholine;

**Table S2.4.2: Assay recovery and plasma matrix effects**

| **Analyte** | **Cer (d18:1/18:0)** | **Cer (d18:1/22:0)** | **Cer (d18:1/24:0)** | **Cer (d18:1/24:1)** | **Cer (d20:1/24:0)** | **Cer (d20:1/24:1)** | **PC (16:0/16:0)** |
| --- | --- | --- | --- | --- | --- | --- | --- |
| **Assay recovery (%)** | | | | | | | |
| **Mean** | 93.92 | 96.48 | 101.97 | 101.08 | 72.39 | 102.00 | 93.75 |
| **%CV** | 5.74 | 2.88 | 6.73 | 5.59 | 5.18 | 8.43 | 19.33 |
| **Matrix effects (%)** | | | | | | | |
| **Mean** | 97.47 | 106.72 | 108.91 | 106.60 | 100.25 | 114.09 | 98.72 |
| **%CV** | 1.01 | 1.84 | 1.52 | 2.69 | 3.20 | 6.02 | 1.22 |

Abbreviations: Cer, Ceramide; PC, phosphatidylcholine; %CV, coefficient of variation

## S2.5 Quality control (QC) samples and stability assessments

QCs were prepared fresh for every analytical run according to the spike concentrations in Table S2.5.1. The spiked concentrations of low-quality control (LQC), medium-quality control (MQC) and high-quality control (HQC) were respectively based on the 20^th^, 50^th^ and 80^th^ percentiles of endogenous plasma concentrations measured by our previously published lipidomic studies.[1, 2, 6] The spiked concentration for upper limit of quantification quality control (ULOQ) was double the 80^th^ percentile value.

The lower limit of quantification quality control (LLOQ) was prepared using a stock solution made at a concentration of 10% of Standard 2 of the calibration spike. Samples were extracted according to usual sample preparation; however, an aqueous solution of 1% bovine serum albumin was used instead of FFP-1.

**Table S2.5.1: Concentration of spiked analytes in quality controls**

| **Lipid species** | **Quality control samples (concentration, mg/L)** | | | | |
| --- | --- | --- | --- | --- | --- |
|  | **Lower Limit of Quantification** | **Low** | **Medium** | **High** | **Upper Limit of Quantification** |
| **Cer(d18:1/18:0)** | 0.005 | 0.05 | 0.064 | 0.074 | 0.149 |
| **Cer(d18:1/22:0)** | 0.075 | 0.714 | 0.857 | 1.429 | 2.857 |
| **Cer(d18:1/24:0)** | 0.130 | 1.857 | 2.429 | 2.857 | 5.714 |
| **Cer(d18:1/24:1)** | 0.105 | 0.857 | 1.143 | 1.429 | 2.857 |
| **Cer(d20:1/24:0)** | 0.002 | 0.017 | 0.026 | 0.029 | 0.057 |
| **Cer(d20:1/24:1)** | 0.002 | 0.011 | 0.014 | 0.02 | 0.04 |
| **PC(16:0/16:0)** | 0.400 | 4.857 | 9.429 | 13.429 | 26.857 |

Abbreviations: Cer, Ceramide; PC, phosphatidylcholine

On-board and in-freezer stability were determined by analysis of freshly extracted QC samples compared to the same samples re-analysed after being stored in the autosampler for 96 hours and in the -20°C freezer for seven days respectively. Mean recovery for on-board stability testing was between 95 – 104% for all species except Cer(d20:1/24:0) where it was 80% (table S2.5.2) and mean recovery for in-freezer stability testing was between 94 – 120% for all species (table S2.5.3).

**Table S2.5.2: On board stability – mean recovery of analytes from LQC, MQC, HQC and ULOQ**

| **Analyte** | **Cer (d18:1/18:0)** | **Cer (d18:1/22:0)** | **Cer (d18:1/24:0)** | **Cer (d18:1/24:1)** | **Cer (d20:1/24:0)** | **Cer (d20:1/24:1)** | **PC (16:0/16:0)** |
| --- | --- | --- | --- | --- | --- | --- | --- |
| **Mean recovery (%)** | 103.48 | 103.48 | 101.43 | 100.56 | 79.61 | 100.83 | 95.42 |
| **Standard deviation** | 3.67 | 2.92 | 7.30 | 2.19 | 8.99 | 5.08 | 13.83 |
| **%CV** | 2.82 | 2.82 | 7.20 | 2.17 | 11.29 | 5.03 | 14.49 |

Abbreviations: Cer, Ceramide; PC, phosphatidylcholine; %CV, coefficient of variation

**Table S2.5.3: In-freezer stability – mean recovery of analytes from LQC, MQC, HQC and ULOQ**

| **Analyte** | **Cer (d18:1/18:0)** | **Cer (d18:1/22:0)** | **Cer (d18:1/24:0)** | **Cer (d18:1/24:1)** | **Cer (d20:1/24:0)** | **Cer (d20:1/24:1)** | **PC (16:0/16:0)** |
| --- | --- | --- | --- | --- | --- | --- | --- |
| **Mean recovery (%)** | 102.67 | 98.91 | 94.97 | 98.54 | 114.21 | 119.30 | 100.56 |
| **Standard deviation** | 8.88 | 8.33 | 6.79 | 7.44 | 11.16 | 16.30 | 8.18 |
| **%CV** | 8.65 | 8.42 | 7.15 | 7.55 | 9.78 | 13.67 | 8.14 |

Abbreviations: Cer, Ceramide; PC, phosphatidylcholine; %CV, coefficient of variation

Stability across three freeze/thaw (F/T) cycles was determined in five human plasma samples. Plasma had been stored for at least five years in a -80°C freezer and had been collected as part of the same trial protocol as the Discovery cohort. This plasma was thawed and 100μL of plasma was extracted according to the standard protocol, and analysed on the LC-MS. The remaining plasma was refrozen at -80°C. This process was repeated twice over the subsequent two weeks. Recovery was calculated as the percentage of the concentration in the first freeze/thawed sample of each of the lipid species in each plasma sample. Mean recovery was calculated using the recovery of each freeze thaw cycle, on each of the plasma samples. Mean recovery was >96% for each analyte (table S2.5.4).

**Table S2.5.4: Mean recovery of analytes from plasma samples across three freeze-thaw cycles**

| **Analyte** | **Cer (d18:1/18:0)** | **Cer (d18:1/22:0)** | **Cer (d18:1/24:0)** | **Cer (d18:1/24:1)** | **Cer (d20:1/24:0)** | **Cer (d20:1/24:1)** | **PC (16:0/16:0)** |
| --- | --- | --- | --- | --- | --- | --- | --- |
| **Mean recovery (%)** | 99.53 | 96.67 | 111.19 | 97.74 | 104.74 | 102.97 | 105.62 |
| **Standard deviation** | 4.45 | 4.68 | 11.55 | 8.57 | 13.18 | 4.23 | 15.59 |
| **%CV** | 4.47 | 4.85 | 10.39 | 8.77 | 12.58 | 4.11 | 14.76 |

Abbreviations: Cer, Ceramide; PC, phosphatidylcholine; %CV, coefficient of variation

## S2.6 Inter- and Intra- assay variation

Inter-assay variation was determined by analysing precision (denoted by percentage coefficient of variation (%CV) of each analyte at the LQC, MQC and HQC over eight run replicates (Table S2.6.1). Intra-assay variation was determined by analysing seven replicates of LQC, MQC and HQC in the same analytical run (Table S2.6.2). Target %CV was below ten percent.[4] CVs were below ten percent for all lipids except Cer(d20:1/24:0) and Cer(d20:1/24:1) where it was <13%. The higher %CV of these two lipids reflects their lower endogenous concentration and remains acceptable as the variables are continuous and used for prognostication.

**Table S2.6.1 Inter-assay variability of analytes in QC samples**

| **Analyte** | **QC sample** | **Mean concentration (mg/L)** | **%CV** | **Expected concentration (mg/L)^^^** | **Accuracy (%)^*^** |
| --- | --- | --- | --- | --- | --- |
| **Cer(d18:1/18:0)** | **LQC** | 0.08 | 3.9 | 0.08 | 100.0 |
|  | **MQC** | 0.11 | 1.8 | 0.094 | 117.0 |
|  | **HQC** | 0.12 | 1.7 | 0.104 | 115.4 |
| **Cer(d18:1/22:0)** | **LQC** | 1.05 | 2.7 | 1.144 | 91.8 |
|  | **MQC** | 1.25 | 3.9 | 1.287 | 97.1 |
|  | **HQC** | 1.87 | 5.1 | 1.859 | 100.6 |
| **Cer(d18:1/24:0)** | **LQC** | 2.95 | 1.9 | 3.112 | 94.8 |
|  | **MQC** | 3.5 | 3.2 | 3.684 | 95.0 |
|  | **HQC** | 3.92 | 2.7 | 4.112 | 95.3 |
| **Cer(d18:1/24:1)** | **LQC** | 1.57 | 3.7 | 1.368 | 114.8 |
|  | **MQC** | 1.94 | 2.6 | 1.654 | 117.3 |
|  | **HQC** | 2.36 | 4.1 | 1.94 | 121.6 |
| **Cer(d20:1/24:0)** | **LQC** | 0.0123 | 11.3 | 0.029 | 42.4 |
|  | **MQC** | 0.0136 | 9.5 | 0.038 | 35.8 |
|  | **HQC** | 0.0144 | 9 | 0.041 | 35.1 |
| **Cer(d20:1/24:1)** | **LQC** | 0.012 | 9.3 | 0.018 | 66.7 |
|  | **MQC** | 0.0126 | 9.7 | 0.021 | 60.0 |
|  | **HQC** | 0.0148 | 12.1 | 0.027 | 54.8 |
| **PC(16:0/16:0)** | **LQC** | 9.86 | 5.7 | 10.427 | 94.6 |
|  | **MQC** | 14.5 | 1.8 | 14.999 | 96.7 |
|  | **HQC** | 17.97 | 4.5 | 18.999 | 94.6 |

Abbreviations: QC, quality control; CV, coefficient of variation; Cer, Ceramide; LQC, low quality control; MQC, middle quality control; HQC, high quality control; PC, phosphatidylcholine

^^^ expected concentration based on FFP-1 plasma concentration of lipid + spiked concentration

* Accuracy = mean concentration/expected concentration * 100

**Table S2.6.2 Intra-assay variability of analytes**

| **Analyte** | **QC sample** | **CV (%)** |
| --- | --- | --- |
| **Cer(d18:1/18:0)** | **LQC** | 2.8 |
|  | **MQC** | 3.4 |
|  | **HQC** | 3 |
| **Cer(d18:1/22:0)** | **LQC** | 3 |
|  | **MQC** | 2.1 |
|  | **HQC** | 2.3 |
| **Cer(d18:1/24:0)** | **LQC** | 4.9 |
|  | **MQC** | 5.7 |
|  | **HQC** | 6.1 |
| **Cer(d18:1/24:1)** | **LQC** | 3 |
|  | **MQC** | 1.4 |
|  | **HQC** | 2 |
| **Cer(d20:1/24:0)** | **LQC** | 10.3 |
|  | **MQC** | 8.6 |
|  | **HQC** | 9 |
| **Cer(d20:1/24:1)** | **LQC** | 6.2 |
|  | **MQC** | 11.4 |
|  | **HQC** | 12.1 |
| **PC(16:0/16:0)** | **LQC** | 7.7 |
|  | **MQC** | 5.6 |
|  | **HQC** | 6.1 |

Abbreviations: QC, quality control; CV, coefficient of variation; Cer, Ceramide; LQC, low quality control; MQC, middle quality control; HQC, high quality control; PC, phosphatidylcholine

## S2.7 Limits of quantification

The signal-to-noise ratio of analytes in LLOQ was greater than 20 for all lipids except for Cer(d20:1/24:1) where it was greater than 10. This is considered acceptable (Table S2.7.1).[4] The %CV for the signal-to-noise ratio of the LLOQ for PC(16:0/16:0) was >10%. As the concentration of the LLOQ for this analyte was well below our target concentration (and was much lower than any of our measured clinical samples), this was considered acceptable.

**Table S2.7.1: LLOQ signal-to-noise ratio, mean value, and coefficient of variation across eight run replicates**

| **Analyte** | **Cer (d18:1/18:0)** | **Cer (d18:1/22:0)** | **Cer (d18:1/24:0)** | **Cer (d18:1/24:1)** | **Cer (d20:1/24:0)** | **Cer (d20:1/24:1)** | **PC (16:0/16:0)** |
| --- | --- | --- | --- | --- | --- | --- | --- |
| **Minimum value signal-to-noise ratio** | 20.16 | 53.21 | 54.31 | 73.58 | 37.67 | 13.69 | 29.55 |
| **Mean (mg/L)** | 0.47 | 1.45 | 1.36 | 0.67 | 0.09486 | 0.1936 | 1.35 |
| **%CV** | 0.98 | 3.96 | 9.48 | 8.66 | 9.45 | 1.97 | 47.21 |

Abbreviations: Cer, Ceramide; PC, phosphatidylcholine; %CV, coefficient of variation

## S2.8 Ion suppression assessment

Ion suppression is assessed via a T-column infusion experiment. An IS-PPS solution of high concentration was directly infused into the mass spectrometer using a syringe pump. Once a stable total ion count was achieved, a sample of FFP (extracted using a PPS without internal standards) was injected concurrently by the HPLC system. If significant ion suppression due to matrix is present, an observable decrease in curve height of the direct infusion will be observed at the time point when the matrix components elute from the column.[4] No attenuation of signal count (i.e., ion suppression) was seen at the time of the peak concentration of any of the ISs. Fluctuations in the signal were observed due to fluctuations in mobile phase concentrations and the use of an older-style pump where small fluctuations reflect alternating pump signals. An initial dip prior to the peaks reflects the bulk of the injection volume (figure S2.8.1A-E).
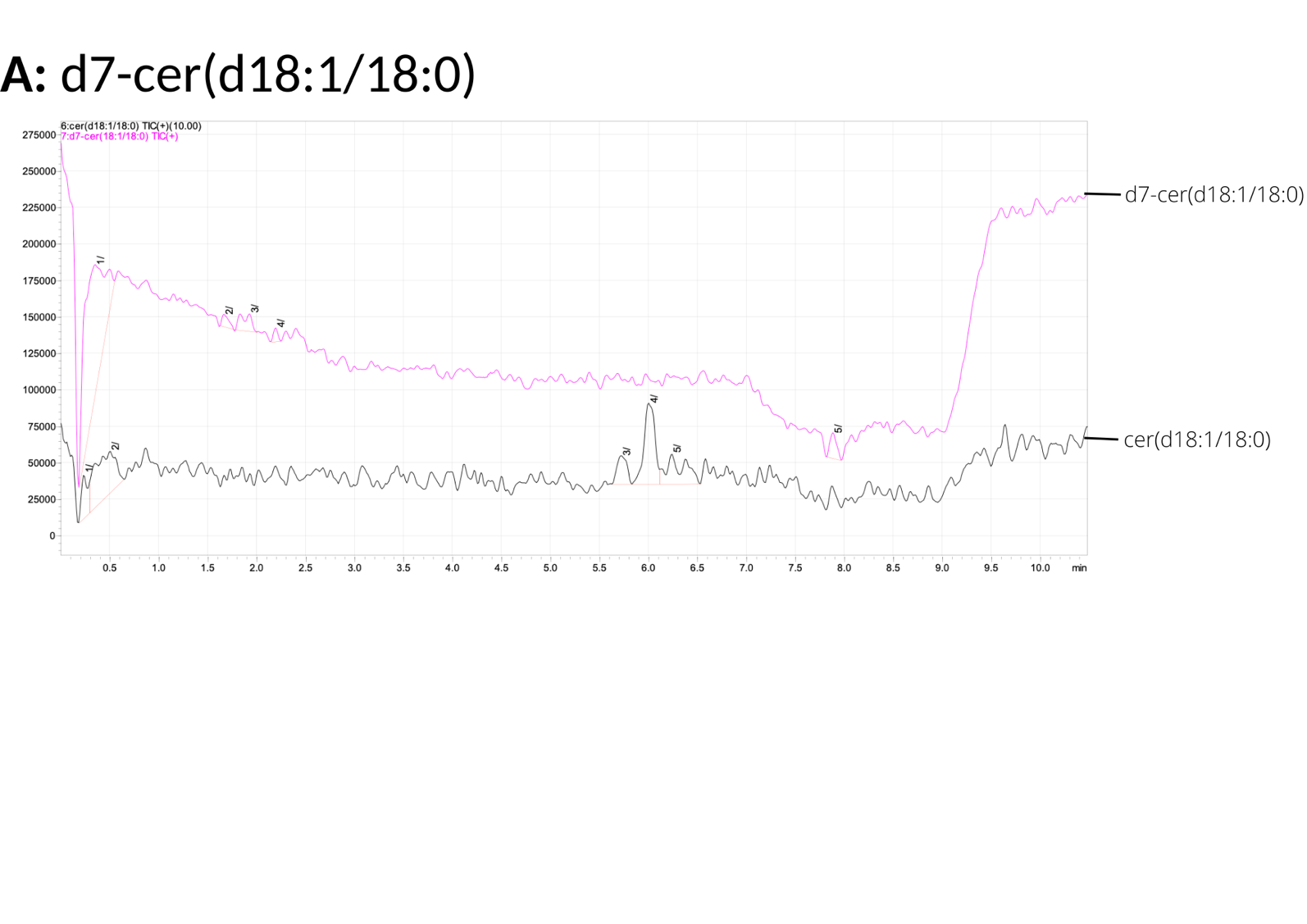


**Figure S2.8.1A Ion suppression assessment: d7-cer(d18:1/18:0)**

Abbreviations: Cer, Ceramide


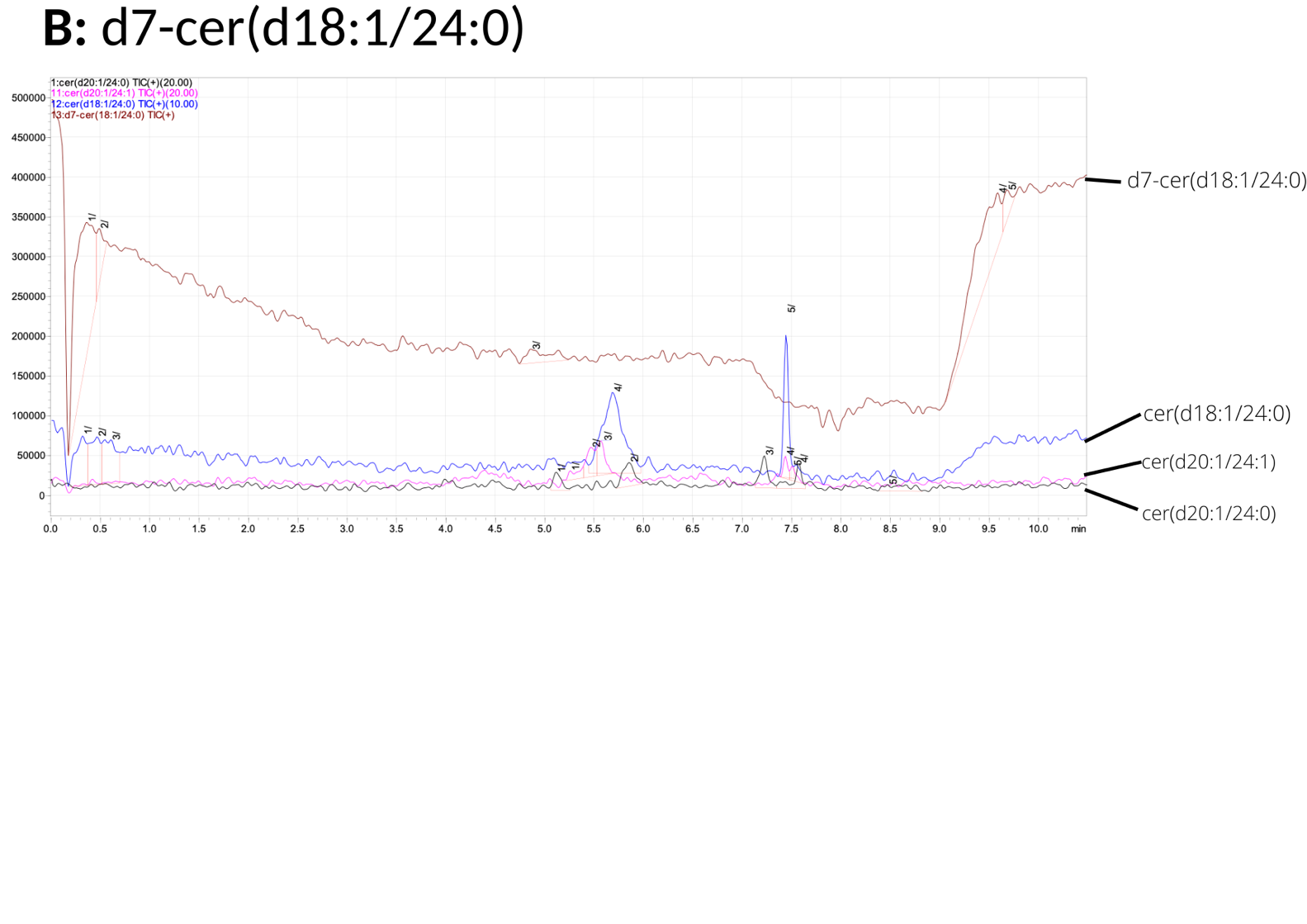


**Figure S2.8.1B Ion suppression assessment: d7-cer(d18:1/24:0)**

Abbreviations: Cer, Ceramide

**
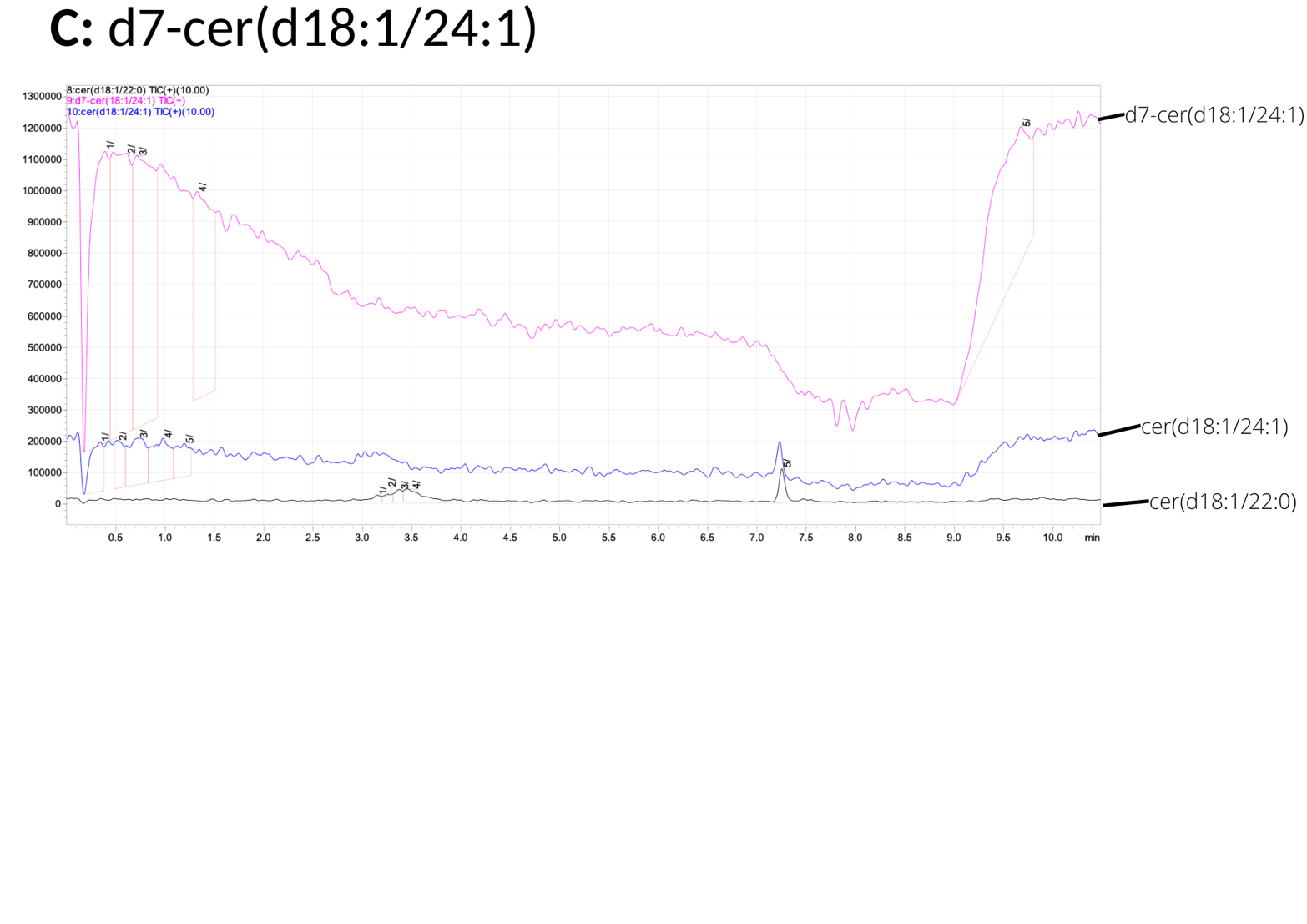
**

**Figure S2.8.1C Ion suppression assessment: d7-cer(d18:1/24:1)**

Abbreviations: Cer, Ceramide

**
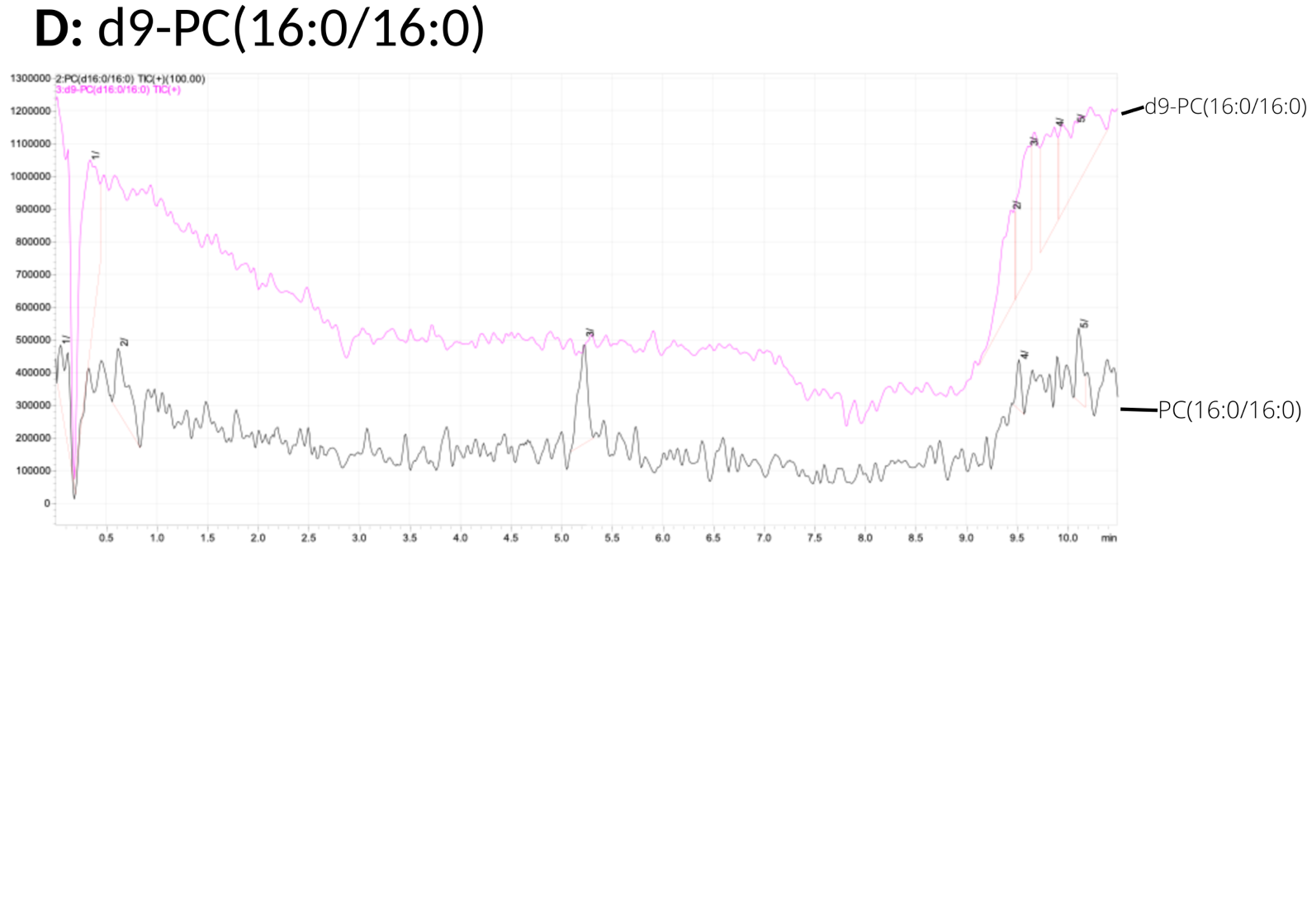
**

**Figure S2.8.1D Ion suppression assessment: d9-PC(16:0/16:0)**

Abbreviations: PC, phosphatidylcholine

**
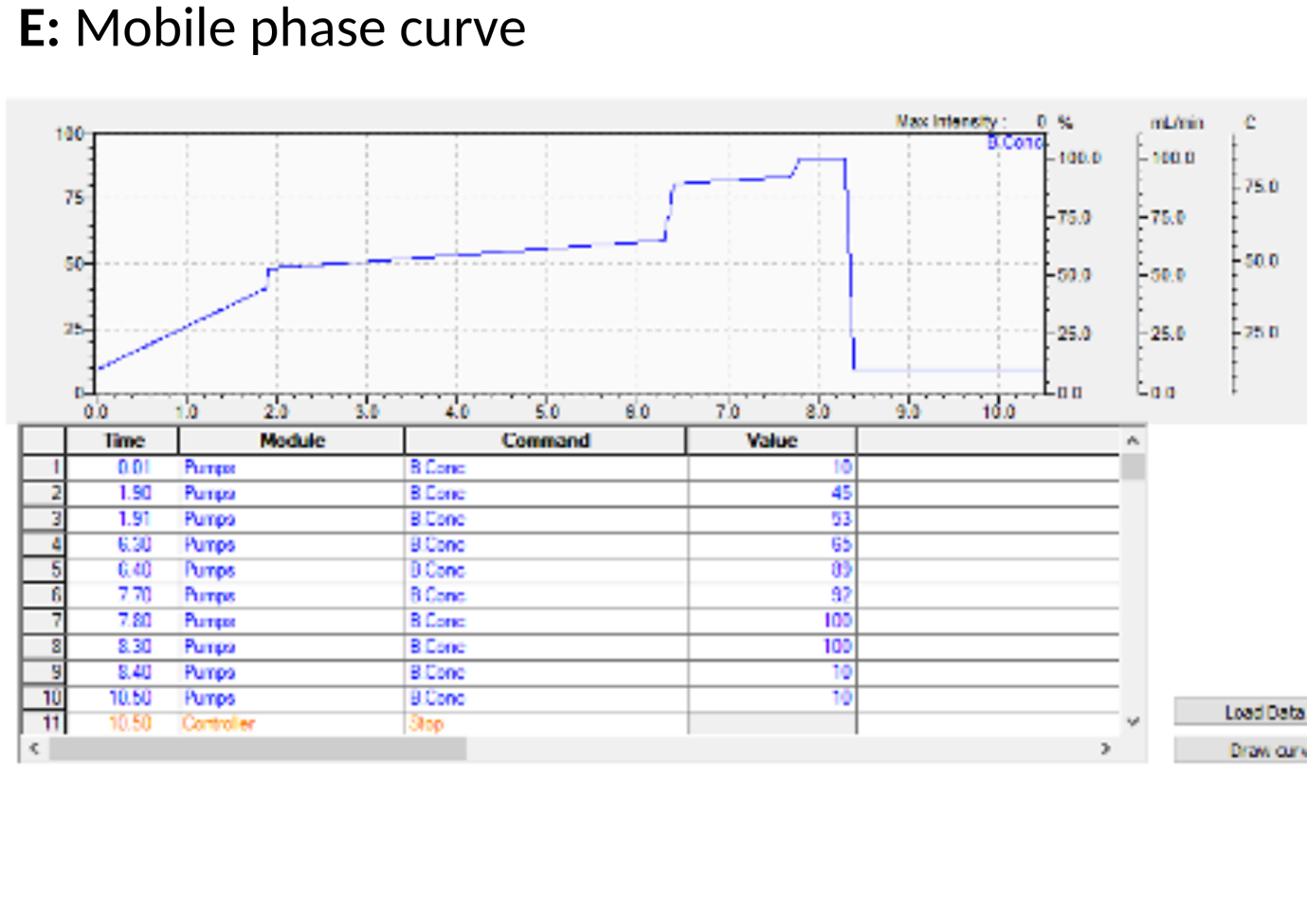
**

**Figure S2.8.1E Ion suppression assessment: Mobile phase curve timing**

Abbreviations: B Conc, Mobile Phase B concentration

## S2.9 Endogenous lipid concentrations in FFP-1

Table S2.9.1 shows the endogenous concentration of lipids in FFP-1 determined by the method of standard addition for the calibration curve.

**Table S2.9.1 Endogenous concentration (mg/L) of each lipid species in blank FFP-1 for each of the eight analytical replicates**

| **Analyte** | **Cer (d18:1/18:0)** | **Cer (d18:1/22:0)** | **Cer (d18:1/24:0)** | **Cer (d18:1/24:1)** | **Cer (d20:1/24:0)** | **Cer (d20:1/24:1)** | **PC (16:0/16:0)** |
| --- | --- | --- | --- | --- | --- | --- | --- |
| **Run #01** | 0.034 | 0.370 | 1.211 | 0.456 | 0.009 | 0.006 | 6.200 |
| **Run #02** | 0.034 | 0.370 | 1.199 | 0.456 | 0.009 | 0.006 | 6.200 |
| **Run #03** | 0.021 | 0.504 | 1.290 | 0.616 | 0.009 | 0.006 | 4.988 |
| **Run #04** | 0.032 | 0.434 | 1.175 | 0.514 | 0.013 | 0.007 | 5.772 |
| **Run #05** | 0.032 | 0.463 | 1.290 | 0.526 | 0.002 (excluded) | 0.007 | 5.114 |
| **Run #06** | 0.028 | 0.404 | 1.239 | 0.48 | 0.010 | 0.008 | 5.139 |
| **Run #07** | 0.028 | 0.463 | 1.290 | 0.543 | 0.025 | 0.006 | 5.253 |
| **Run #08** | 0.031 | 0.430 | 1.346 | 0.498 | 0.007 | 0.008 | 5.436 |
| **Mean concentration (mg/L)** | 0.030 | 0.430 | 1.255 | 0.511 | 0.012 | 0.007 | 5.570 |
| **Standard deviation** | 0.00 | 0.05 | 0.06 | 0.05 | 0.01 | 0.00 | 0.49 |
| **%CV** | 14.17 | 10.97 | 4.63 | 10.32 | 53.44 | 13.13 | 8.83 |

Abbreviations: Cer, Ceramide; PC, phosphatidylcholine; %CV, coefficient of variation

# S3. High-throughput plasma lipidomic analysis

## S3.1 High-throughput plasma lipidomic analysis method

Plasma lipidomic analysis for the Discovery cohort was performed in the study described in Lin *et al* (2017)[1], using the methods described in Weir et al (2013)[7] with minor modifications outlined in Lin et al (2017). Lipid extraction was performed with chloroform/methanol and the LC-MS platform used was an Agilent 1200 liquid chromatography system with an Applied Biosystems API 4000 Q/TRAP mass spectrometer.

Plasma lipidomic analysis for the Validation cohort was performed in the study described in Lin et al (2021),[2] using the methods described in Huynh et al (2019).[8] A butanol/methanol lipid extraction method was used, whereas the LC-MS platform was an Agilent 1290 series HPLC system with an Agilent 6490 QQQ mass spectrometer.

For the Discovery cohort, 323 lipid species from 22 lipid classes/subclasses were quantified, whereas for the Validation cohort, 824 lipid species from 47 lipid classes were quantified. In both studies, the concentration of lipid species was calculated by comparison with relevant internal standards and adjusted with response factors. The data was then normalised independently using the Probabilistic Quotient normalisation method as described previously[1]. The original normalised data were used for comparison to the data from the targeted assays.

## S3.2. Calculation of the three-lipid signature (3LS)

Determination of the 3LS status in the Discovery and Validation cohorts was performed in the respective lipidomic studies [1, 2] using the formula below which was derived and displayed in Lin *et al* (2017)[1]:


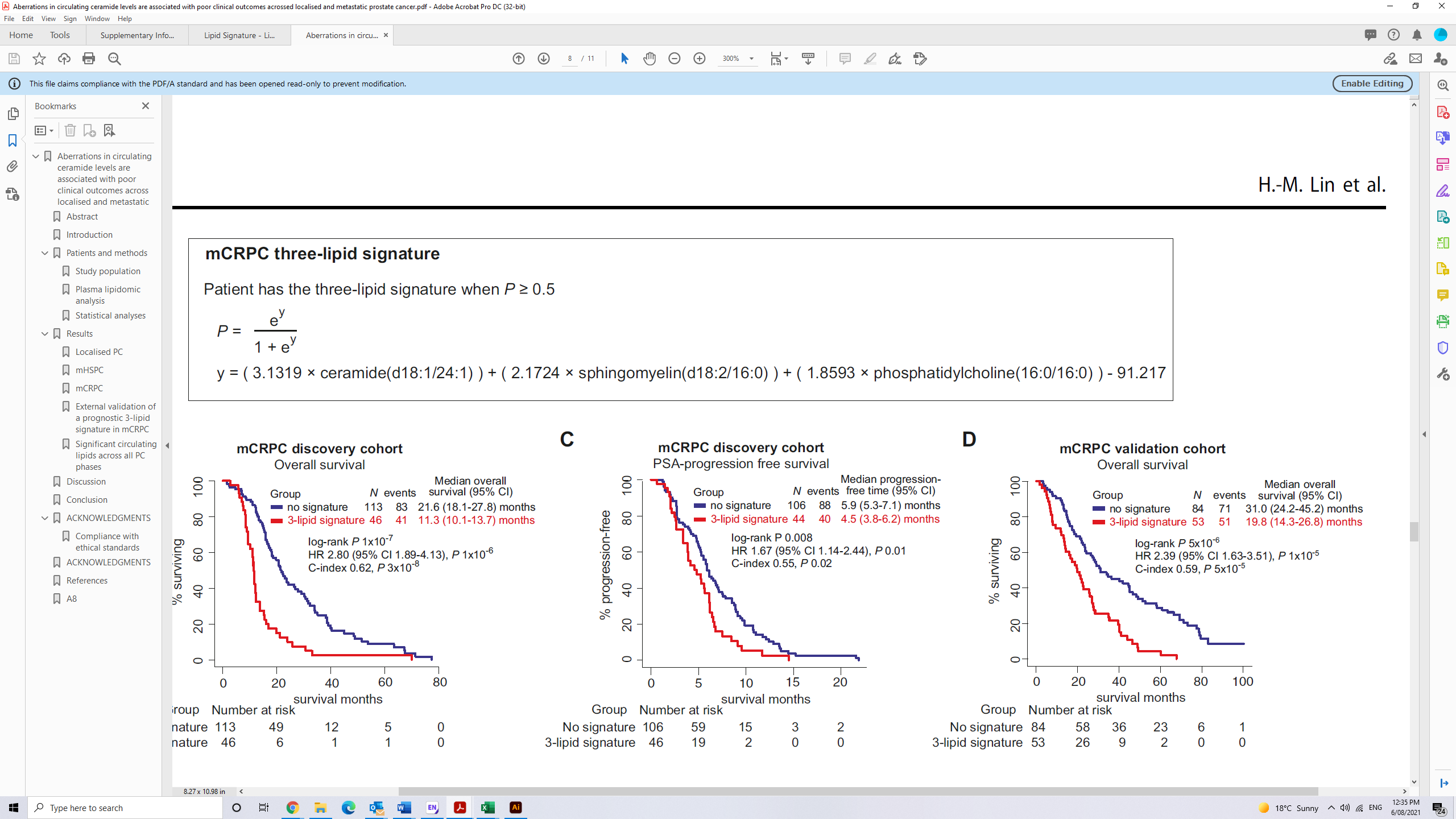


As described in Lin et al (2021),[2] determination of the 3LS status for the Validation cohort required the alignment of the normalised data to the original cohort in Lin *et al* (2017) prior to calculation with the formula above. This data alignment accounted for LC-MS platform and batch differences and was performed using the ComBat algorithm (R package sva, v3.34.0).

# S4. References for statistical analysis methods

## S4.1 R statistical software environment v4.1.1

R Core Team (2022). R: A language and environment for statistical computing. R Foundation for Statistical Computing, Vienna, Austria. URL <https://www.R-project.org/>.

**Pearson’s Correlation coefficient**

R package ‘ggplot2; v3.3.5

Wilkinson L. ggplot2: Elegant Graphics for Data Analysis by WICKHAM, H. *Biometrics* 2011; **67**(2): 678-679; doi 10.1111/j.1541-0420.2011.01616.x.

**Univariable Cox Regression**

R package ‘survival’ v3.2-13

Andersen PK, Gill RD. Cox's Regression Model for Counting Processes: A Large Sample Study. *The Annals of statistics* 1982; **10**(4): 1100-1120; doi 10.1214/aos/1176345976.

Therneau TM, Grambsch PM. *Modeling Survival Data: Extending the Cox Model*. Springer New York: New York, NY, 2000.

**Least absolute shrinkage and selection operator (LASSO)**

R package ‘glmnet’ v4.1-2

Tibshirani R. THE LASSO METHOD FOR VARIABLE SELECTION IN THE COX MODEL. *Statistics in medicine* 1997; **16**(4): 385-395; doi 10.1002/(SICI)1097-0258(19970228)16:4<385::AID-SIM380>3.0.CO;2-3.

**Weibull regression model**

R package ‘survival’ v3.2-13

Kalbfleisch JD, Prentice RL. *The statistical analysis of failure time data*, 2nd ed. ed. J. Wiley: Hoboken, N.J, 2002.

**Residuals analysis**

R package ‘survival’ v3.2-13

Andersen PK, Gill RD. Cox's Regression Model for Counting Processes: A Large Sample Study. *The Annals of statistics* 1982; **10**(4): 1100-1120; doi 10.1214/aos/1176345976.

Therneau TM, Grambsch PM. *Modeling Survival Data: Extending the Cox Model*. Springer New York: New York, NY, 2000.

## S4.2 IBM SPSS v27

*IBM SPSS Statistics, Version 27.0* [computer program]. Armonk, NY: IBM Corp; Released 2020.

#
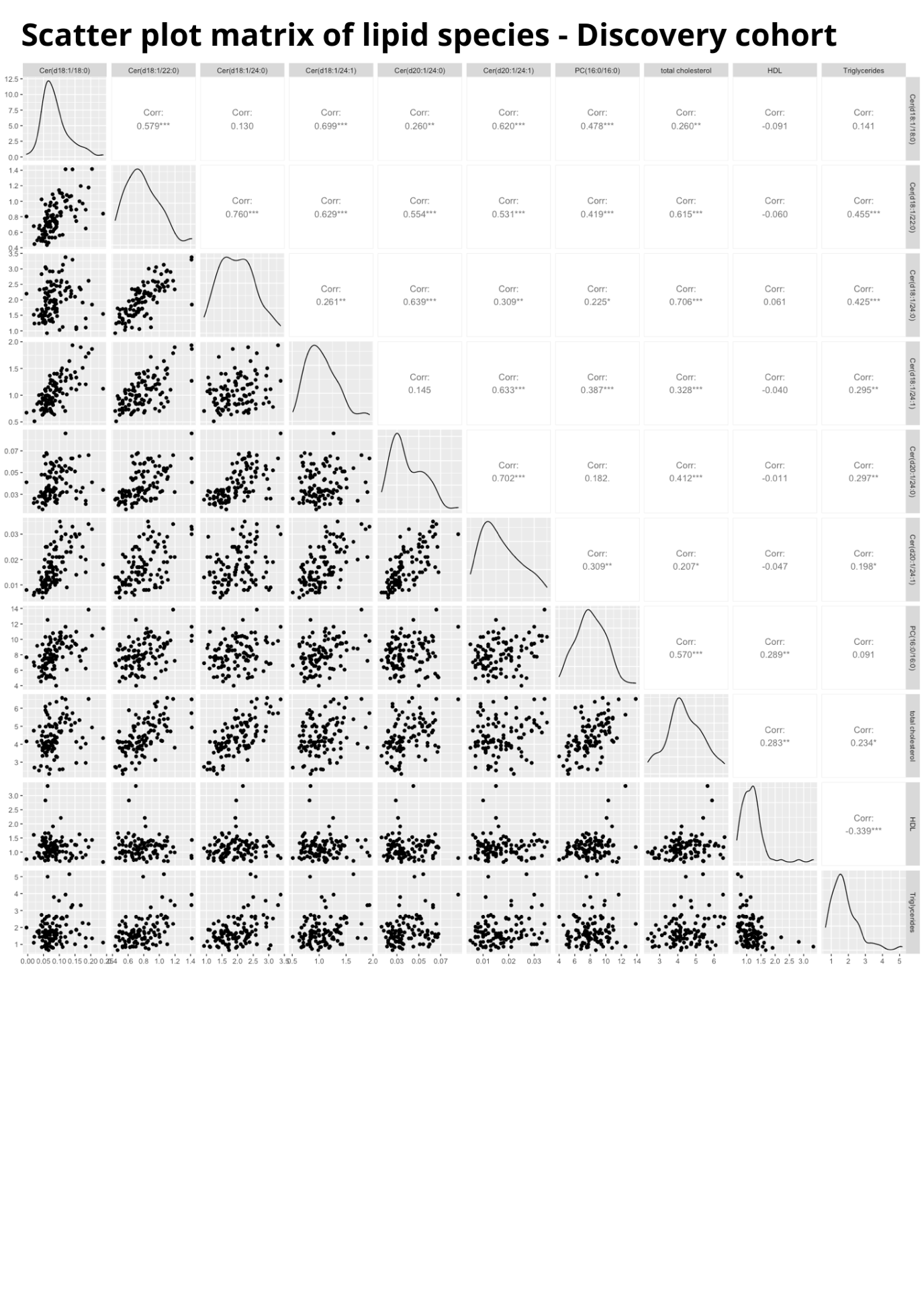
S5. Correlation matrix of lipid concentrations measured by the targeted assay

**Figure S5.1 Correlation matrix of the concentrations between pairs of lipid species measured by the targeted assay on the Discovery cohort. Scatterplots display concentrations of individual samples. “Corr” values are Pearson’s correlation coefficient. Line plots on the diagonal axis display the distribution of concentrations of each analyte.**

Abbreviations: Cer, ceramide; PC, phosphatidylcholine; HDL, high density lipoprotein; Corr., Pearson’s *R*


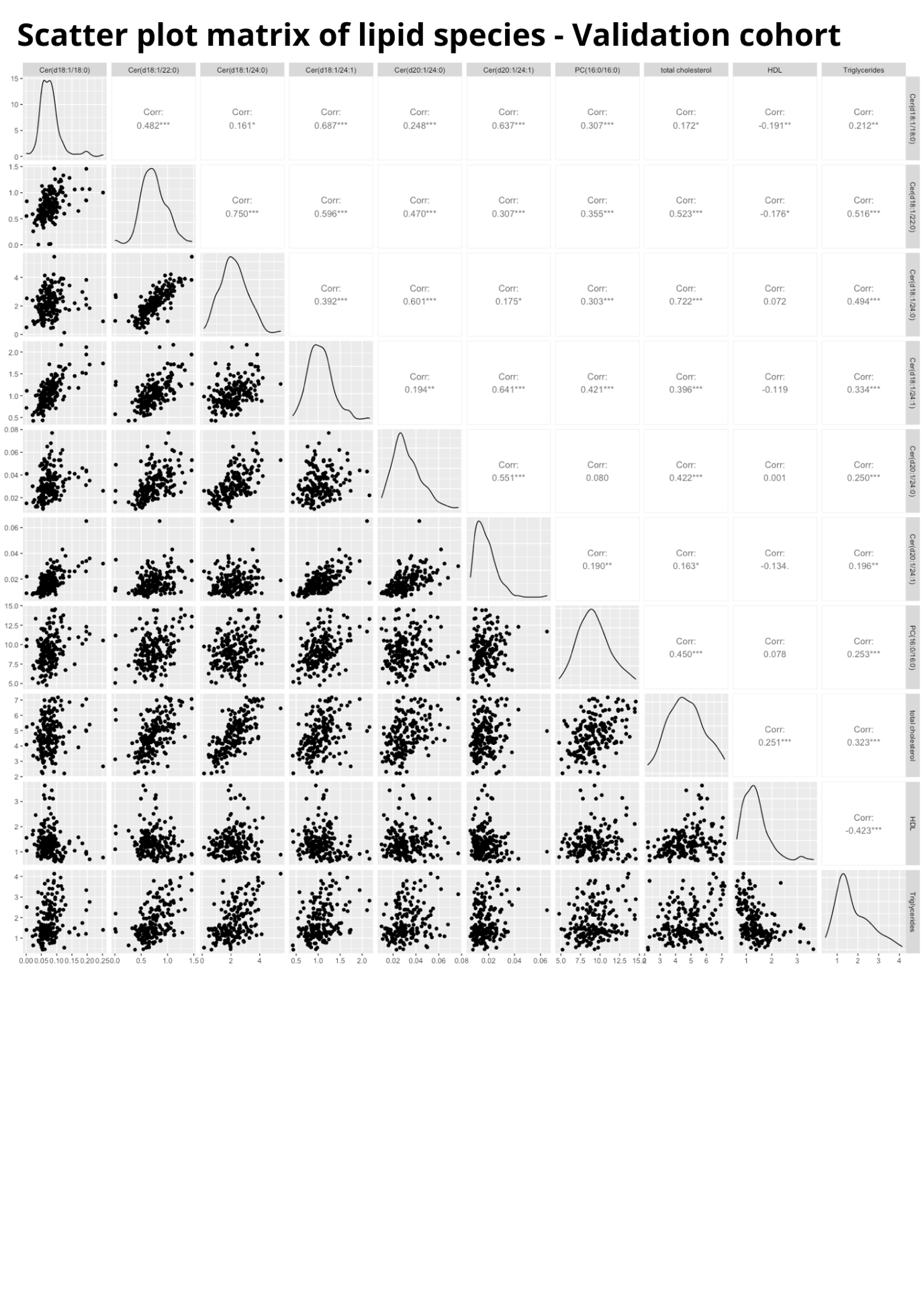


**Figure S5.2. Correlation matrix of the concentrations between pairs of lipid species measured by the targeted assay on the Validation cohort. Scatterplots display concentrations of individual samples. “Corr” values are Pearson’s correlation coefficient. Line plots on the diagonal axis display the distribution of concentrations of each analyte.**

Abbreviations: Cer, ceramide; PC, phosphatidylcholine; HDL, high density lipoprotein; Corr., Pearson’s *R*

# S6. Models developed by Cox Regression with the LASSO shrinkage method

**Model 1:**

This model includes all the lipids measured via the targeted LC-MS assay and the clinical enzymatic assay.

**Model inputs:**

*Mg/L:* Cer(d18:1/18:0), Cer(d18:1/22:0), Cer(d18:1/24:0), Cer(d18:1/24:1), Cer(d20:1/24:0), Cer(d20:1/24:1), PC(16:0/16:0)

*Mmol/L:* total cholesterol, HDL, triglycerides

**Model post LASSO shrinkage:**

| *Σβx* = (−0.241 x Cer(d18:1/24:0)) + (12.07 x Cer(d18:1/18:0)) + (−0.2657 x total cholesterol) + (−0.2164 x triglycerides) |
| --- |

**Model 2:**

This model includes only the lipids measured via the targeted LC-MS assay, for ease of clinical use, in order to minimise steps required to formally run PCPro.

**Model inputs:**

*Mg/L:* Cer(d18:1/18:0), Cer(d18:1/22:0), Cer(d18:1/24:0), Cer(d18:1/24:1), Cer(d20:1/24:0), Cer(d20:1/24:1), PC(16:0/16:0)

**Model post LASSO shrinkage:**

| *Σβx* = (−0.6597 x Cer(d18:1/24:0)) + (9.2732 x Cer(d18:1/18:0)) + (10.5265 x Cer(d20:1/24:1)) |
| --- |

**Model 3:**

This model includes all the lipids from model 1, however Cer(d18:1/24:0) has been included as higher levels of this ceramide are associated with better prognosis, whereas higher levels of the other ceramides are associated with worse prognosis, and we wanted to assess performance of this model without this lipid.

**Model inputs:**

*Mg/L:* Cer(d18:1/18:0), Cer(d18:1/22:0), Cer(d18:1/24:1), Cer(d20:1/24:0), Cer(d20:1/24:1), PC(16:0/16:0)

*Mmol/L:* total cholesterol, HDL, triglycerides

**Model post LASSO shrinkage:**

| *Σβx* = (0.4434 x Cer(d18:1/24:1)) + (-0.8446 x Cer(d18:1/22:0) + (12.2 x Cer(d18:1/18:0)) + (−0.2957 x total cholesterol) + (−0.2128 x triglycerides) |
| --- |

**Model 4:**

This model contains just the lipids measured via clinical enzymatic assay, in order to assess the performance of the model created via conventional, widely available parameters.

**Model inputs:**

*Mmol/L:* total cholesterol, HDL, triglycerides

**Model post LASSO shrinkage:**

| *Σβx* = (−0.1209 x total cholesterol) + (−0.2398 x HDL) + (-0.3013 x triglycerides) |
| --- |

**Model 5:**

This model contains all the lipids of model 1, as well as the ratio between Cer(d18:1/24:0) and Cer(d18:1/24:1). These variables are inversely correlated with survival, and their inclusion adjusts for confounding effects of the co-regulation of their metabolism.

**Model inputs:**

*Mg/L:* Cer(d18:1/18:0), Cer(d18:1/22:0), Cer(d18:1/24:0), Cer(d18:1/24:1), Cer(d20:1/24:0), Cer(d20:1/24:1), PC(16:0/16:0)

*Mmol/L:* total cholesterol, HDL, triglycerides

*Ratio:* Cer(d18:1/24:0)/Cer(d18:1/24:1)

**Model post LASSO shrinkage:**

| *Σβx* = (10.0506 x Cer(d18:1/18:0)) + (−0.2783 x total cholesterol) + (−0.2240 x triglycerides) + (-0.2979 x (Cer(d18:1/24:0)/Cer(d18:1/24:1))) |
| --- |

**Model 6:**

This model contains all the lipids of model 1, as well as the difference between Cer(d18:1/24:0) and Cer(d18:1/24:1). These variables are inversely correlated with survival, and their inclusion adjusts for confounding effects of the co-regulation of their metabolism.

**Model inputs:**

*Mg/L:* Cer(d18:1/18:0), Cer(d18:1/22:0), Cer(d18:1/24:0), Cer(d18:1/24:1), Cer(d20:1/24:0), Cer(d20:1/24:1), PC(16:0/16:0)

*Mmol/L:* total cholesterol, HDL, triglycerides

*Difference (mg/L):* Cer(d18:1/24:0) - Cer(d18:1/24:1)

**Model post LASSO shrinkage:**

| *Σβx* = (10.9903 x Cer(d18:1/18:0)) + (−0.2779 x total cholesterol) + (−0.2243 x triglycerides) + (-0.2366 x (Cer(d18:1/24:0) - Cer(d18:1/24:1))) |
| --- |

**Model 7:**

This model contains all the lipids of model 1, as well as both the ratio and the difference between Cer(d18:1/24:0) and Cer(d18:1/24:1). These variables are inversely correlated with survival, and their inclusion adjusts for confounding effects of the co-regulation of their metabolism.

**Model inputs:**

*Mg/L:* Cer(d18:1/18:0), Cer(d18:1/22:0), Cer(d18:1/24:0), Cer(d18:1/24:1), Cer(d20:1/24:0), Cer(d20:1/24:1), PC(16:0/16:0)

*Mmol/L:* total cholesterol, HDL, triglycerides

*Ratio:*  Cer(d18:1/24:0)/Cer(d18:1/24:1)

*Difference (mg/L):* Cer(d18:1/24:0) - Cer(d18:1/24:1)

**Model post LASSO shrinkage:**

| *Σβx* = (10.0506 x Cer(d18:1/18:0)) + (−0.2783 x total cholesterol) + (−0.2240 x triglycerides) + (-0.2979 x (Cer(d18:1/24:0)/Cer(d18:1/24:1))) |
| --- |

**Model 8:**

This model contains all the lipids of model 5, however triglycerides have been removed. Triglycerides are the lipid that is likely to be affected most by fasting status, and performance of the model was evaluated without this lipid.

**Model inputs:**

*Mg/L:* Cer(d18:1/18:0), Cer(d18:1/22:0), Cer(d18:1/24:0), Cer(d18:1/24:1), Cer(d20:1/24:0), Cer(d20:1/24:1), PC(16:0/16:0)

*Mmol/L***:** total cholesterol, HDL

*Ratio:* Cer(d18:1/24:0)/Cer(d18:1/24:1)

**Model post LASSO shrinkage:**

| *Σβx* = (9.6096 x Cer(d18:1/18:0)) + (−0.2863 x total cholesterol) + (−0.3543 x (Cer(d18:1/24:0)/Cer(d18:1/24:1))) |
| --- |

**Model 9:**

This model contains all of the lipids of model 1, as well as the ratio of each ceramide to total ceramides (as measured by the sum of the lipids measured via targeted LC-MS assay). There is large amounts of co-regulation of ceramide synthesis and metabolism, and ceramide ratios were used to overcome some of this confounding factor.

**Model inputs:**

*Mg/L:* Cer(d18:1/18:0), Cer(d18:1/22:0), Cer(d18:1/24:0), Cer(d18:1/24:1), Cer(d20:1/24:0), Cer(d20:1/24:1), PC(16:0/16:0)

*Mmol/L:* total cholesterol, HDL, triglycerides

*Ratio:* Cer(d18:1/18:0)/total ceramide*, Cer(d18:1/22:0)/total ceramide*, Cer(d18:1/24:0)/total ceramide*, Cer(d18:1/24:1)/total ceramide*, Cer(d20:1/24:0)/total ceramide*, Cer(d20:1/24:1)/total ceramide*

* Total ceramide = Cer(d18:1/18:0) + Cer(d18:1/22:0) + Cer(d18:1/24:0) + Cer(d18:1/24:1) + Cer(d20:1/24:0) + Cer(d20:1/24:1)

**Model post LASSO shrinkage:**

| *Σβx* = (-0.1579 x total cholesterol) + (−1.949 x Cer(d18:1/24:0)/total ceramide) + (43.74 x Cer(d18:1/18:0)/total ceramide) |
| --- |

**Model 10:**

This model used all the lipids and ratios of model 9, as well as the difference between Cer(d18:1/24:0) and Cer(d18:1/24:1), as a further measure to overcome the co-regulation of ceramide synthesis and metabolism.

**Model inputs:**

*Mg/L:* Cer(d18:1/18:0), Cer(d18:1/22:0), Cer(d18:1/24:0), Cer(d18:1/24:1), Cer(d20:1/24:0), Cer(d20:1/24:1), PC(16:0/16:0)

*Mmol/L:* total cholesterol, HDL, triglycerides

*Ratio:* Cer(d18:1/18:0)/total ceramide*, Cer(d18:1/22:0)/total ceramide*, Cer(d18:1/24:0)/total ceramide*, Cer(d18:1/24:1)/total ceramide*, Cer(d20:1/24:0)/total ceramide*, Cer(d20:1/24:1)/total ceramide*, Cer(d18:1/24:0)/Cer(d18:1/24:1)

*Difference (mg/L):* Cer(d18:1/24:0) - Cer(d18:1/24:1)

* Total ceramide = Cer(d18:1/18:0) + Cer(d18:1/22:0) + Cer(d18:1/24:0) + Cer(d18:1/24:1) + Cer(d20:1/24:0) + Cer(d20:1/24:1)

**Model post LASSO shrinkage:**

| *Σβx* = (42.01 x Cer(d18:1/18:0)/total ceramide) + (-0.3235 x (Cer(d18:1/24:0)/Cer(d18:1/24:1))) |
| --- |

**Model 11:**

This model includes the lipids of model 1, as well as the ratio of each of the ceramides to total cholesterol (as measured on the clinical enzymatic assay). Ceramide and cholesterol synthesis, metabolism and transportation, are co-regulated, and this model was used to overcome these confounding effects.

**Model inputs:**

*Mg/L:* Cer(d18:1/18:0), Cer(d18:1/22:0), Cer(d18:1/24:0), Cer(d18:1/24:1), Cer(d20:1/24:0), Cer(d20:1/24:1), PC(16:0/16:0)

*Mmol/L:* total cholesterol, HDL, triglycerides

*Ratio:* Cer(d18:1/18:0)/total cholesterol, Cer(d18:1/22:0)/total cholesterol, Cer(d18:1/24:0)/total cholesterol, Cer(d18:1/24:1)/total cholesterol, Cer(d20:1/24:0)/total cholesterol, Cer(d20:1/24:1)/total cholesterol

**Model post LASSO shrinkage:**

| *Σβx* = (33.84 x (Cer(d18:1/18:0)/total cholesterol) + (1.59 x (Cer(d18:1/24:1)/total cholesterol) + (0.3304 x (PC(16:0/16:0)/total cholesterol)) |
| --- |

**Model 12:**

This model includes all the lipids of model 1, as well as all the ratios from model 5, model 9 and model 11, as well as the difference between Cer(d18:1/24:0) and Cer(d18:1/24:1). This model included all the possible iterations of models 1 – 11, for the reasons explained above.

**Model inputs:**

*Mg/L:* Cer(d18:1/18:0), Cer(d18:1/22:0), Cer(d18:1/24:0), Cer(d18:1/24:1), Cer(d20:1/24:0), Cer(d20:1/24:1), PC(16:0/16:0)

*Mmol/L:* total cholesterol, HDL, triglycerides

*Ratio:* Cer(d18:1/18:0)/total ceramide*, Cer(d18:1/22:0)/total ceramide*, Cer(d18:1/24:0)/total ceramide*, Cer(d18:1/24:1)/total ceramide*, Cer(d20:1/24:0)/total ceramide*, Cer(d20:1/24:1)/total ceramide*, Cer(d18:1/18:0)/total cholesterol, Cer(d18:1/22:0)/total cholesterol, Cer(d18:1/24:0)/total cholesterol, Cer(d18:1/24:1)/total cholesterol, Cer(d20:1/24:0)/total cholesterol, Cer(d20:1/24:1)/total cholesterol, Cer(d18:1/24:0)/Cer(d18:1/24:1)

*Difference (mg/L):* Cer(d18:1/24:0) - Cer(d18:1/24:1)

* Total ceramide = Cer(d18:1/18:0) + Cer(d18:1/22:0) + Cer(d18:1/24:0) + Cer(d18:1/24:1) + Cer(d20:1/24:0) + Cer(d20:1/24:1)

**Model post LASSO shrinkage:**

| *Σβx* = (-0.1495 x total cholesterol) + (49.98 x (Cer(d18:1/18:0)/total ceramide)) + (-8.275 x (Cer(d18:1/18:0)/total cholesterol) + (0.1557 x (PC(16:0/16:0)/total cholesterol) + (-0.2464 x (Cer(d18:1/24:0)/Cer(d18:1/24:1))) |
| --- |

# S7. Optimal cut-point determination

## S7.1 Cut-points tested for Model 5

**Table S7.1.1 Univariate Cox regression and Weibull regression analysis of good and poor prognosis groups formed by different cut-points, which range from the median to the 70th percentile scores of Model 5.**

| **Cut-point** | **Univariate Cox regression HR [95% CI]** | **Concordance** | **Weibull parametric regression log-likelihood** |
| --- | --- | --- | --- |
| -1.3809 (median) | 2.42 [1.55 - 3.77] | 0.606 | -345.0 |
| -1.3622 | 2.31 [1.48 – 3.59] | 0.597 | -345.8 |
| -1.3362 | 2.70 [1.70 – 4.27] | 0.606 | -343.3 |
| -1.3178 | 2.56 [1.62 – 4.05] | 0.597 | -344.2 |
| -1.2942 | 2.80 [1.76 – 4.46] | 0.605 | -342.9 |
| -1.2888 | 2.76 [1.74 – 4.38] | 0.605 | -343.3 |
| -1.2828 | 2.96 [1.85 – 4.73] | 0.611 | -342.2 |
| -1.281 | 2.96 [1.85 – 4.71] | 0.613 | -342.4 |
| -1.2711 | 2.89 [1.82 – 4.60] | 0.612 | -343.0 |
| -1.2607 | 2.88 [1.81 – 4.58] | 0.613 | -343.2 |
| -1.2340 | 2.93 [1.84 – 4.66] | 0.616 | -343.2 |
| -1.2293 | 2.97 [1.87 – 4.73] | 0.618 | -343.3 |
| -1.2219 | 3.03 [1.90 – 4.84] | 0.621 | -343.2 |
| -1.2198 | 3.72 [2.27 – 6.10] | 0.629 | -340.3 |
| -1.1953 | 3.73 [2.28 – 6.12] | 0.630 | -340.2 |
| -1.1903 | 3.75 [2.29 – 6.15] | 0.631 | -340.1 |
| -1.1885 | 3.67 [2.24 – 6.01] | 0.629 | -340.6 |
| -1.181 | 3.60 [2.20 – 5.89] | 0.626 | -341.2 |
| -1.1708 | 3.61 [2.21 – 5.90] | 0.627 | -341.2 |
| -1.1669 | 3.71 [2.26 – 6.08] | 0.629 | -341.2 |
| -1.166 (worst 30%) | 3.80 [2.31 – 6.26] | 0.630 | -341.3 |

*Shaded cell is the optimal cut-off (i.e. the highest C-statistic (concordance) and Weibull parametric regression log-likelihood)

Abbreviations: HR, hazard ratio; CI, confidence interval

## S7.2 Cut-points tested for Model 6

**Table S7.2.1 Univariate Cox regression and Weibull regression analysis of good and poor prognosis groups formed by different cut-points, which range from the median to the 70th percentile scores of Model 6.**

| **Cut-point** | **Univariate Cox regression HR [95% CI]** | **Concordance** | **Weibull parametric regression log-likelihood** |
| --- | --- | --- | --- |
| -0.926 (median) | 2.74 [1.72 – 4.35] | 0.603 | -343.1 |
| -0.914 | 2.77 [1.74 – 4.39] | 0.608 | -342.8 |
| -0.913 | 3.03 [1.90 – 4.85] | 0.615 | -341.3 |
| -0.912 | 2.98 [1.87 – 4.75] | 0.616 | -341.7 |
| -0.906 | 2.97 [1.87 – 4.72] | 0.617 | -342.0 |
| -0.861 | 2.99 [1.89 – 4.76] | 0.620 | -342.0 |
| -0.8397 | 2.85 [1.79 – 4.51] | 0.611 | -343.0 |
| -0.8396 | 2.78 [1.76 – 4.41] | 0.610 | -343.5 |
| -0.832 | 3.00 [1.88 – 4.78] | 0.616 | -342.4 |
| -0.8238 | 3.01 [1.89 – 4.79] | 0.617 | -342.3 |
| -0.8237 | 3.05 [1.92 – 4.86] | 0.620 | -342.2 |
| -0.817 | 3.71 [2.26 – 6.08] | 0.628 | -339.3 |
| -0.808 | 3.63 [2.21 – 5.94] | 0.627 | -339.9 |
| -0.785 | 3.54 [2.16 – 5.78] | 0.624 | -340.5 |
| -0.766 | 3.51 [2.15 – 5.73] | 0.624 | -341.0 |
| -0.755 | 3.53 [2.16 – 5.77] | 0.625 | -341.2 |
| -0.77 | 3.65 [2.23 – 5.97] | 0.627 | -341.2 |
| -0.762 | 3.63 [2.21 – 5.95] | 0.626 | -341.7 |
| -0.76 | 3.64 [2.22 – 5.97] | 0.627 | -341.5 |
| -0.757 | 3.58 [2.18 – 5.87] | 0.625 | -342.1 |
| -0.75 (worst 30%) | 3.672 [2.23 – 6.04] | 0.626 | -342.2 |

*Shaded cell is the optimal cut-off (i.e. the highest C-statistic (concordance) and Weibull parametric regression log-likelihood)

Abbreviations: HR, hazard ratio; CI, confidence interval

# S8. Verification of Cox proportional hazards assumptions for PCPro by Residuals testing in the Discovery Cohort

## S8.1 Schoenfeld residuals

The relationship between residuals and time is non-significant for the individual variables or the global model of PCPro, thus proportional hazards can be assumed for the individual variables and the global model (table S8.1.1, figure S8.1.1).

**Table S8.1.1 Schoenfeld residual test for variables included in the final model of PCPro**

| **Variable** | **Chi-square** | **df** | **P value** |
| --- | --- | --- | --- |
| Cer(d18:1/18:0) | 0.4 | 1 | 0.5 |
| Cholesterol | 0.1 | 1 | 0.8 |
| Triglycerides | 1.1 | 1 | 0.3 |
| Cer(d18:1/24:0)/Cer(d18:1/24:1) | 2.3 | 1 | 0.1 |
| **GLOBAL** | 3.3 | 4 | 0.5 |

Abbreviations: df, degrees of freedom; Cer, ceramide


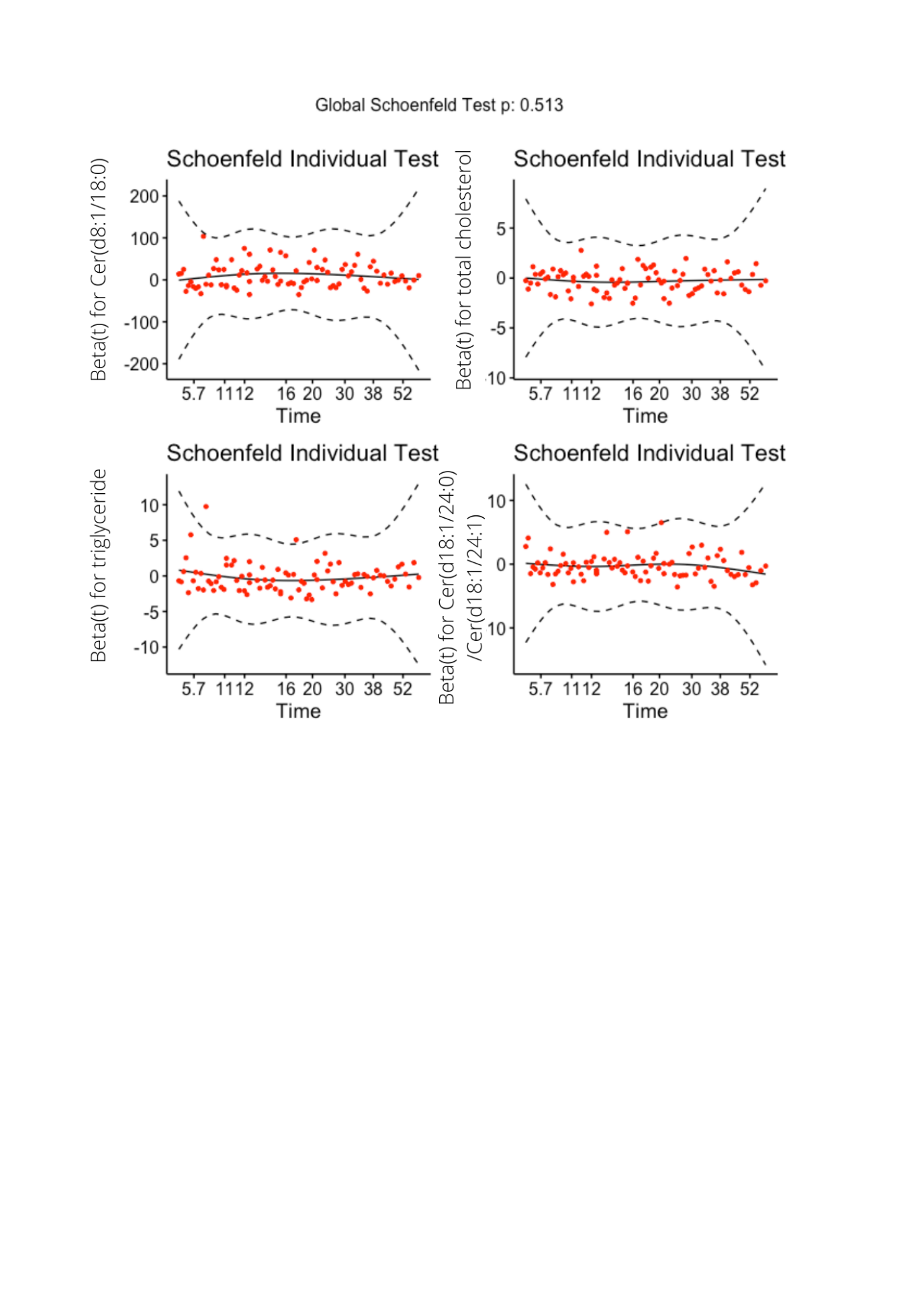


**Figure S8.1.1 Schoenfeld tests for variables included in the final model of PCPro**

## S8.2 Martingale residuals

Plots of Martingale residuals against the continuous variables of PCPro were generally linear or more linear than their transformed variables (logarithm, or square root). Thus, linearity of the variables in the model can be assumed, and their transformation was not necessary (figure S8.2.1).


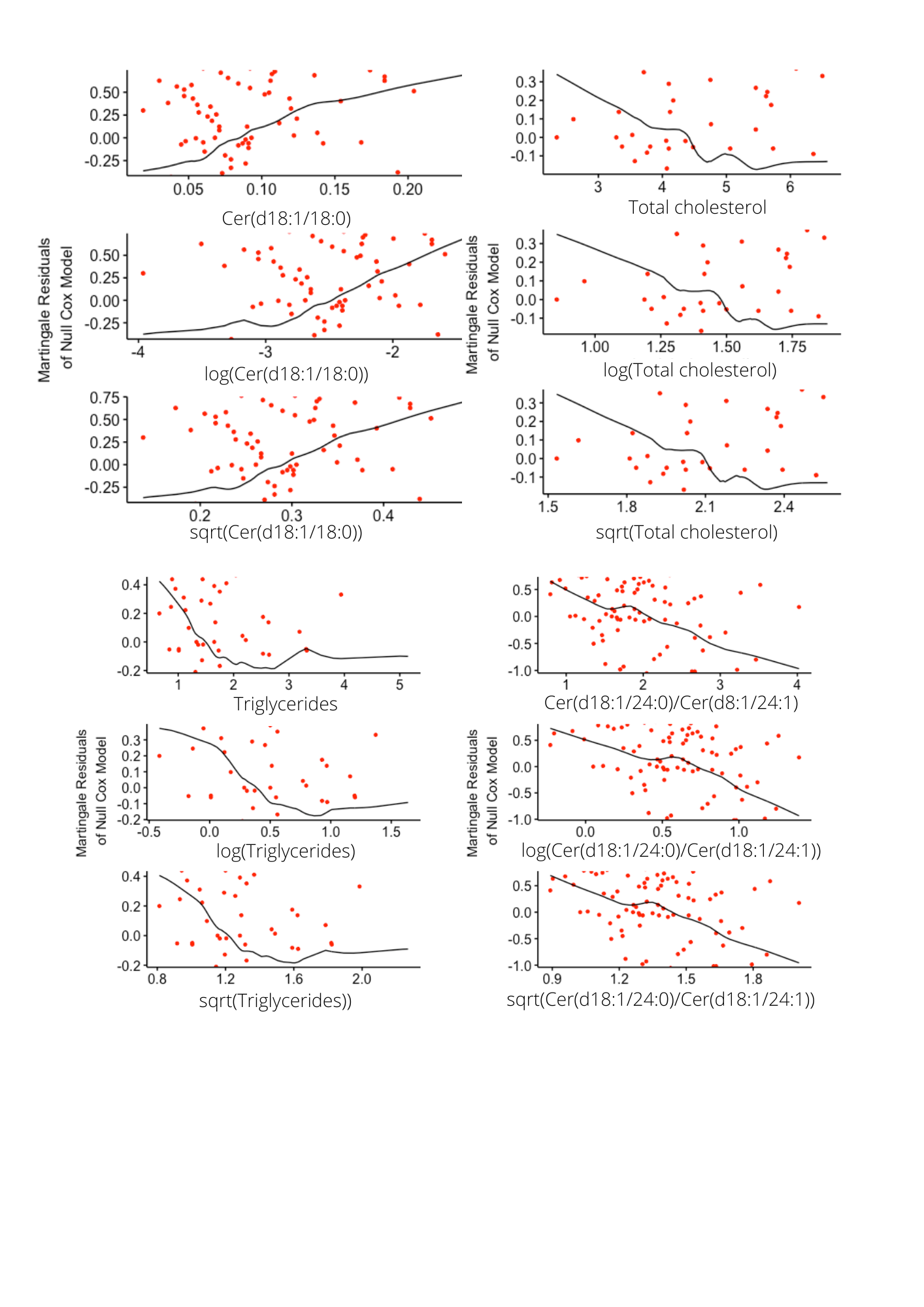


**Figure S8.2.1: Martingale Residuals of null Cox models for untransformed, log transformed, and square root transformed variables in PCPro**

#
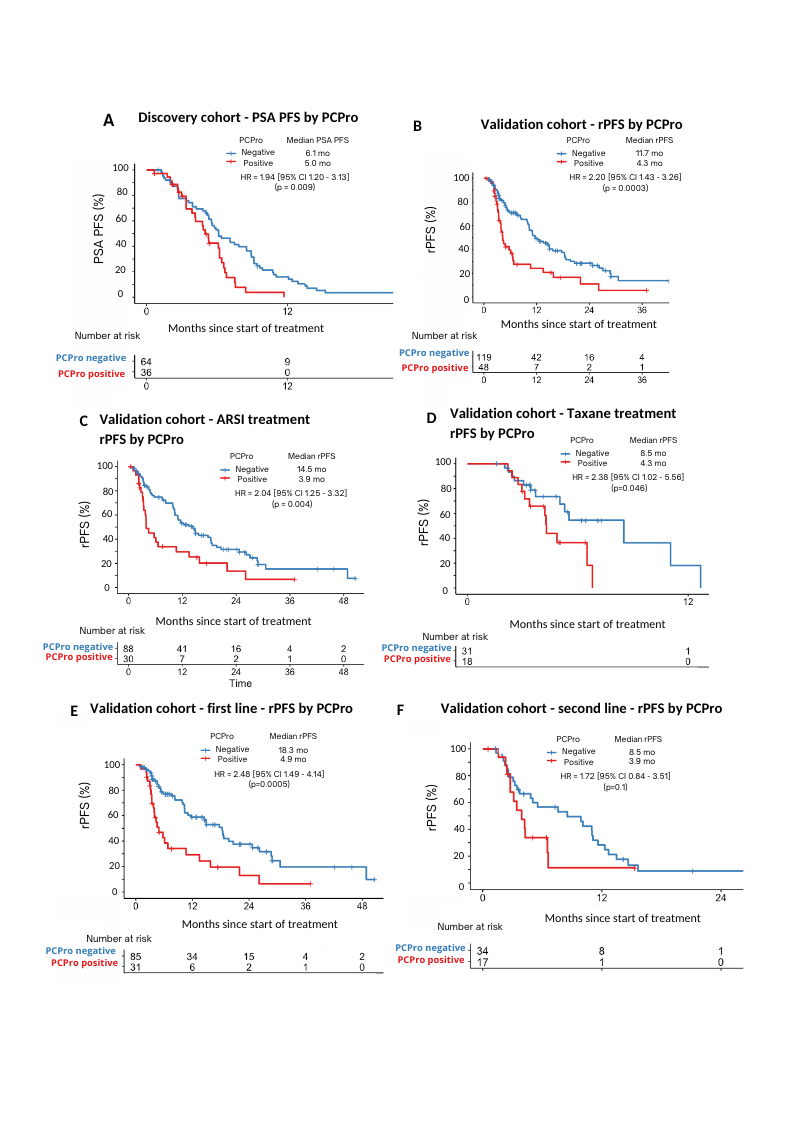
S9. Kaplan-Meier Survival analysis of progression free survival (PFS) by PCPro in the Discovery and Validation cohorts

**Figure S9.1 Kaplan-Meier plots of progression free survival (PFS) in (A) the Discovery cohort, (B) the Validation cohort, (C), those treated with an ARSI in the Validation cohort, (D), those treated with taxane chemotherapy in the Validation cohort, (E), those treated with first line treatment in the Validation cohort and (F) those treated with second line therapy in the Validation cohort. All the plots are of rPFS except for the Discovery cohort (A) as rPFS data was not available.**

Abbreviations: PSA, prostate specific antigen; PFS, progression free survival; rPFS, radiographic progression free survival; HR, hazard ratio; mo, months; ARSI, androgen receptor signalling inhibitor

# S10. Correlation of PCPro with the three-lipid prognostic signature

## S10.1 Correlation in the Discovery cohort


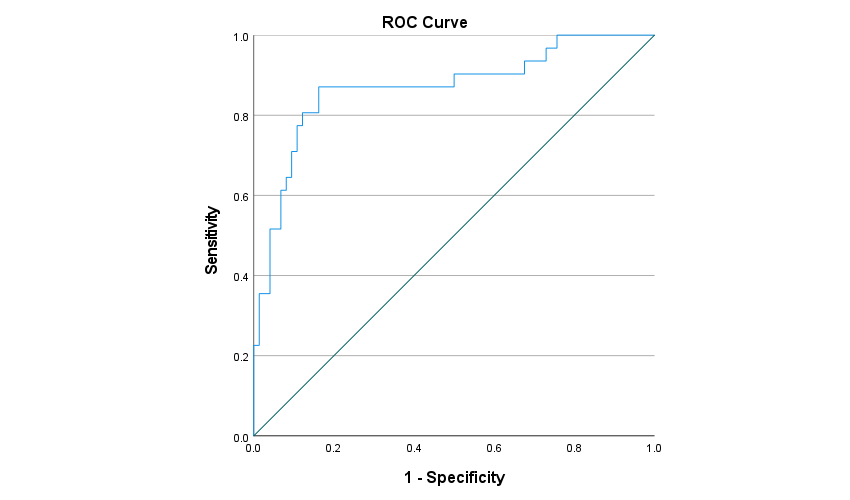


**Figure S10.1.1: Receiver Operator Characteristic (ROC) curve of PCPro compared to the three-lipid signature in the Discovery cohort, where ROC area under the curve (AUC) = 0.869**

Abbreviations: ROC, receiver operator characteristic

**Table S10.1.1: Cox regression and survival analysis of 3LS and PCPro in the Discovery cohort**

| **Model (Discovery cohort)** | **Risk group** | **Cases** | **Median overall survival, months** | **Cox regression of risk groups** | |
| --- | --- | --- | --- | --- | --- |
|  |  |  |  | **HR [95% CI]** | **p-value** |
| 3LS | Low-risk | 74 | 23.7 | 3.94 [2.38 – 6.53] | <0.001 |
|  | High-risk | 31 | 11.5 |  |  |
| PCPro | Negative | 64 | 26.4 | 3.63 [2.21 – 5.94] | <0.001 |
|  | Positive | 41 | 12.2 |  |  |

Abbreviations: CI, confidence interval; HR, hazard ratio; 3LS, three-lipid signature

## S10.2 Correlation in the Validation cohort


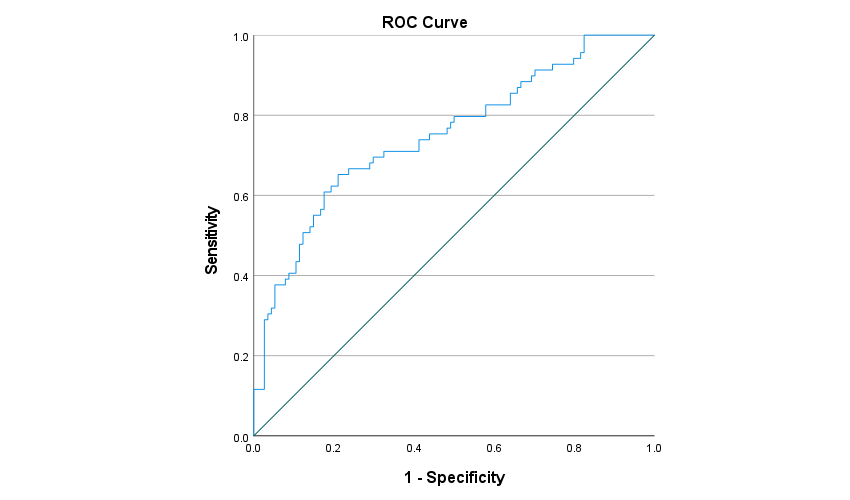


**Figure S10.2.1: Receiver Operator Characteristic (ROC) curve of PCPro compared to the three-lipid signature in the Validation cohort, where ROC area under the curve (AUC) = 0.751**

Abbreviations: ROC, receiver operator characteristic

**Table S10.2.1: Cox regression and survival analyses of 3LS and PCPro in the Validation cohort**

| **Model (Validation cohort)** | **Risk group** | **Cases** | **Median overall survival, months** | **Cox regression of risk groups** | |
| --- | --- | --- | --- | --- | --- |
|  |  |  |  | **HR [95% CI]** | **p-value** |
| 3LS | Low-risk | 114 | 25.0 | 1.96 [1.34 – 2.85] | <0.001 |
|  | High-risk | 69 | 16.1 |  |  |
| PCPro | Negative | 131 | 27.2 | 2.38 [1.60 – 3.53] | <0.001 |
|  | Positive | 52 | 13.8 |  |  |

Abbreviations: CI, confidence interval; HR, hazard ratio; 3LS, three-lipid signature

# S11. Comparison of PCPro to clinicopathological factors

## S11.1 Cox Regression Analysis with clinicopathological factors

### Discovery cohort

**Table S11.1.1: Univariable and multivariable Cox regression analyses of PCPro and clinicopathological factors, discovery cohort (only significant variables taken through to multivariable analysis)**

**(**Neutrophil to lymphocyte ratio information and albumin was not available for the Discovery cohort**)**

| **Variable*** | **Univariable analysis** | | **Multivariable analysis** | |
| --- | --- | --- | --- | --- |
|  | **Hazard ratio [95% CI]** | **P-value** | **Hazard ratio [95% CI]** | **P-value** |
| PCPro (positive vs. negative) | 3.75 [2.29 – 6.15] | <0.001*** | 3.19 [1.90 – 5.35] | <0.001*** |
| Alkaline phosphatase (U/L) | 1.00 [1.00 – 1.00] | 0.005*** | 1.00 [1.00 – 1.00] | 0.025*** |
| Haemoglobin (g/L) | 0.98 [0.96 – 0.99] | 0.003*** | 0.99 [0.97 – 1.00] | 0.075 |
| Serum PSA (μg/L) | 1.00 [1.00 – 1.00] | 0.6 |  |  |
| Metastasis site (bone only/none vs. soft tissue)** | 1.17 [0.75 – 1.82] | 0.5 |  |  |

* Analysed as a continuous variable unless stated otherwise

** Visceral and nodal metastases were not distinguished in our database in the Discovery cohort

*** P value statistically significant (p≤0.05)

Abbreviations: CI = confidence interval, PSA = Prostate specific antigen

### Validation cohort

**Table S11.1.2: Univariable and multivariable Cox regression analyses of PCPro and clinicopathological factors, validation cohort (only significant variables taken through to multivariable analysis)**

| **Variable*** | **Univariate analysis** | | **Multivariable analysis** | |
| --- | --- | --- | --- | --- |
|  | **Hazard ratio [95% CI]** | **P-value** | **Hazard ratio [95% CI]** | **P-value** |
| PCPro (positive vs. negative) | 2.37 [1.59 – 3.52] | <0.001*** | 1.86 [1.20 – 2.88] | 0.006*** |
| Alkaline phosphatase (U/L) | 1.00 [1.00 – 1.00] | 0.03*** | 1.00 [1.00 – 1.00] | 0.6 |
| Haemoglobin (g/L) | 0.98 [0.97 – 0.99] | <0.001*** | 0.99 [0.97 – 1.00] | 0.021*** |
| Serum PSA (μg/L) | 1.00 [1.00 – 1.00] | 0.05*** | 1.00 [1.00 – 1.00] | 0.4 |
| Albumin (g/L) | 0.90 [0.87 – 0.94] | <0.001*** | 0.93 [0.89 – 0.98] | 0.003*** |
| Metastasis site (bone only/lymph node/none vs. visceral) | 1.46 [0.78 – 2.73] | 0.2 |  |  |
| Neutrophil to lymphocyte ratio | 1.04 [0.99 – 1.08] | 0.1 |  |  |

* Analysed as a continuous variable unless stated otherwise

*** P value statistically significant (p≤0.05)

CI = confidence interval, PSA = Prostate specific antigen

## S11.2 Comparison of PCPro to Halabi’s nomogram and a clinicopathological model

Halabi’s nomogram was accessed at: https://www.cancer.duke.edu/Nomogram/firstlinechemotherapy.html[9]. Some of the clinical variables for Halabi’s nomogram were unavailable (Discovery cohort: lactate dehydrogenase levels unavailable for all patients; ECOG performance status, albumin levels, and opiate usage only available for some patients; Validation cohort: data on opiate usage unavailable for all patients; lactate dehydrogenase levels, ECOG performance status, albumin levels only available for some patients). Missing values were estimated as described in the table footnotes below. For additional comparison utilising complete clinicopathological data, another clinicopathological model was derived from multivariable Cox regression of existing data, also described in the table footnotes below.

### Discovery cohort

**Table S11.2.1 Survival and Cox regression analyses of risk groups defined by Halabi’s nomogram, a clinicopathological model and PCPro in the Discovery cohort**

| **Model (Discovery cohort)** | **Risk group** | **Cases** | **Median overall survival, months** | **Cox regression of risk groups** | |
| --- | --- | --- | --- | --- | --- |
|  |  |  |  | **HR [95% CI]** | **P-value** |
| Halabi’s nomogram * | Low-risk | 32 | 27.8 | 1.94 [1.17 – 3.20] | 0.009 |
|  | High-risk | 70 | 15.8 |  |  |
| Clinicopathological model (ALP, Hb, PSA, metastasis site)** | Low-risk | 66 | 21.2 | 2.10 [1.33 – 3.31] | 0.001 |
|  | High-risk | 37 | 12.2 |  |  |
| PCPro | Negative | 67 | 24.2 | 3.75 [2.29 – 6.15] | <0.001 |
|  | Positive | 38 | 12.0 |  |  |

* Lactate dehydrogenase values were unavailable and thus were assumed to be below the normal limit for all patients. Missing values for ECOG performance status, albumin levels and opiate use were estimated by the k-nearest neighbours’ method with a Euclidean metric, using the module “ImputeMissingValuesKNN” in GenePattern version 3.2.3. Number of neighbours used in the imputation was 10.

**The clinicopathological model is the prognostic index (*B1X1* + *B2X2* + *B3X3 +*.... + *BnXn*) of multivariable Cox regression of alkaline phosphatase (ALP), haemoglobin (Hb), PSA, and metastasis site (Table S11.2.2). The size of the risk groups is based on the same proportion as for PCPro.

Abbreviations: HR, hazard ratio; CI, confidence interval; ALP, alkaline phosphatase; Hb, haemoglobin; PSA, prostate specific antigen

**Table S11.2.2 Regression coefficients of the clinicopathological model in table S11.2.1 (Discovery cohort, 105 cases)**

| **Variable** | **Regression coefficient (B)** | **HR [95% CI]** | **p-value** |
| --- | --- | --- | --- |
| **Alkaline phosphatase (U/L)** | 0.00056620 | 1.00 [1.00 – 1.00] | 0.020 |
| **Haemoglobin (U/L)** | -0.0246 | 0.98 [0.96 – 0.99] | 0.006 |
| **PSA (μg/L)** | -0.00001845 | 1.00 [1.00 – 1.00] | 0.8 |
| **Metastatic site (bone only/none vs soft tissue)*** | 0.1421 | 1.15 [0.73 – 1.81] | 0.5 |

* Nodal metastases were not distinguished from visceral metastases in our database.

Abbreviations: HR, hazard ratio; CI, confidence interval; PSA, prostate specific antigen

### Validation cohort

**Table S11.2.3 Survival and Cox regression analyses of risk groups defined by Halabi’s nomogram, a clinicopathological model and PCPro in the Validation cohort**

| **Model (Validation cohort)** | **Risk group** | **Cases** | **Median overall survival, months** | **Cox regression of risk groups** | |
| --- | --- | --- | --- | --- | --- |
|  |  |  |  | **HR [95% CI]** | **P-value** |
| Halabi’s nomogram * | Low-risk | 105 | 27.2 | 2.29 [1.57 – 3.34] | <0.001 |
|  | High-risk | 78 | 13.8 |  |  |
| Clinicopathological model (ALP, Hb, PSA, metastasis site, neutrophil to lymphocyte ratio, albumin)** | Low-risk | 132 | 25.0 | 2.77 [1.87 – 4.10] | <0.001 |
|  | High-risk | 49 | 10.3 |  |  |
| PCPro | Negative | 133 | 25.7 | 2.37 [1.59 – 3.52] | <0.001 |
|  | Positive | 50 | 13.0 |  |  |

* Opioid usage data was unavailable and was assumed to be “no usage”. Lactate dehydrogenase values were only available for some patients and were assumed to be below the normal limit for all other patients. Missing values for ECOG performance status and albumin levels were estimated by the k-nearest neighbours’ method with a Euclidean metric, using the module “ImputeMissingValuesKNN” in GenePattern version 3.2.3. Number of neighbours used in the imputation was 10.

**The clinicopathological model is the prognostic index (*B1X1* + *B2X2* + *B3X3 +*.... + *BnXn*) of multivariable Cox regression of alkaline phosphatase (ALP), haemoglobin (Hb), PSA, metastasis site, neutrophil to lymphocyte ratio and albumin (Table S11.2.4). The size of the risk groups is based on the same proportion as for PCPro. 2 patients did not have neutrophil to lymphocyte ratio available and these patients were excluded from this model.

Abbreviations: HR, hazard ratio; CI, confidence interval; ALP, alkaline phosphatase; Hb, haemoglobin; PSA, prostate specific antigen

**Table S11.2.4 Regression coefficients of the clinicopathological model in table S11.2.3 (Validation cohort, 183 cases)**

| **Variable** | **Regression coefficient (B)** | **HR [95% CI]** | **p-value** |
| --- | --- | --- | --- |
| **Alkaline phosphatase (U/L)** | 0.0001615 | 1.00 [1.00 – 1.00] | 0.2 |
| **Haemoglobin (U/L)** | -0.0136874 | 0.99 [0.97 – 1.00] | 0.03 |
| **PSA (μg/L)** | 0.0002768 | 1.00 [1.00 – 1.00] | 0.1 |
| **Metastatic site (bone only, lymph node, none vs. visceral)** | 0.2650894 | 1.30 [0.71 – 2.45] | 0.4 |
| **Neutrophil to lymphocyte ratio** | 0.0190749 | 1.02 [0.97 – 1.07] | 0.4 |
| **Albumin (g/L)** | -0.0773415 | 0.93 [0.89 – 0.97] | <0.001 |

Abbreviations: HR, hazard ratio; CI, confidence interval; PSA, prostate specific antigen

## S11.3 Relationship between PCPro and diabetic status in the Discovery cohort

**Table S11.3.1 Chi-square test of PCPro and diabetic status in the Discovery cohort**

| **Discovery cohort** | | **Number of cases** | | **Chi-square test p-value** |
| --- | --- | --- | --- | --- |
|  |  | **PCPro negative** | **PCPro positive** |  |
| **Diabetes status*** | **Diagnosed** | 10 | 5 | 0.5 |
|  | **Not diagnosed** | 50 | 36 |  |

*Diabetes status unavailable for 4 patients (all PCPro negative)

**Table S11.3.2 Univariable and bivariable Cox regression of diabetic status and PCPro in the Discovery cohort**

| **Variable*** | **Univariable Cox regression** | | **Bivariable Cox regression** | |
| --- | --- | --- | --- | --- |
|  | **Hazard ratio [95% CI]** | **p-value** | **Hazard ratio [95% CI]** | **p-value** |
| **Diabetes (diagnosed/not diagnosed)** | 1.08 [0.57 – 2.05] | 0.8 | 1.13 [0.59 – 2.15] | 0.7 |
| **PCPro (positive/negative)** | 3.79 [2.29 – 6.28] | <0.001 | 3.80 [2.29 – 6.30] | <0.001 |

*Only included patients who have diabetic status available

Abbreviations: CI, confidence interval

Diabetic status was not available for the Validation cohort.

## S11.4 Univariable and bivariable Cox Regression of age and PCPro

### S11.4.1 Univariable and bivariable Cox Regression of age and PCPro in the Discovery Cohort

**Table S11.4.1.1 Univariable and bivariable Cox regression of age and PCPro in the Discovery cohort**

| **Variable** | **Univariable Cox regression** | | **Bivariable Cox regression** | |
| --- | --- | --- | --- | --- |
|  | **Hazard ratio [95% CI]** | **p-value** | **Hazard ratio [95% CI]** | **p-value** |
| **Age*** | 0.99 [0.9634 – 1.02] | 0.5 | 0.98 [0.96 – 1.01] | 0.3 |
| **PCPro (positive/negative)** | 3.75 [2.29 – 6.15] | <0.001 | 3.88 [2.36 – 6.38] | <0.001 |

^*^Age is a continuous variable

### S11.4.2 Univariable and bivariable Cox Regression of age and PCPro in the Validation Cohort

**Table S11.4.2.1 Univariable and bivariable Cox regression of age and PCPro in the Discovery cohort**

| **Variable** | **Univariable Cox regression** | | **Bivariable Cox regression** | |
| --- | --- | --- | --- | --- |
|  | **Hazard ratio [95% CI]** | **p-value** | **Hazard ratio [95% CI]** | **p-value** |
| **Age*** | 0.99 [0.99 – 1.03] | 0.3 | 1.01 [0.99 – 1.03] | 0.4 |
| **PCPro (positive/negative)** | 2.37 [1.59 – 3.52] | <0.001 | 2.38 [1.60 – 3.56] | <0.001 |

^*^Age is a continuous variable

## S11.5 Univariable Cox regression of traditional cardiovascular risk factors and PCPro

**Table S11.5.1 Univariable Cox regression of traditional cardiovascular risk factors and PCPro in the Discovery cohort**

| **Variable** | **Univariable Cox regression** | |
| --- | --- | --- |
|  | **Hazard ratio [95% CI]** | **p-value** |
| **Weight* (kg)** | 1.00 [0.98 – 1.01] | 0.8 |
| **BMI* (kg/m^2^)** | 1.00 [0.95 – 1.06] | 0.9 |
| **Statins use (use/non-use)** | 1.21 [0.75 – 1.97] | 0.4 |
| **PCPro (positive/negative)** | 3.75 [2.29 – 6.15] | <0.001 |

^*^Weight and BMI are continuous variables

Data on these risk factors were not available in the Validation cohort.

## S11.6 Relationship between PCPro and statin use in the Discovery cohort

**Table S11.6.1 Chi-square test of PCPro and statin use in the Discovery cohort**

| **Discovery cohort** | | **Number of cases** | | **Chi-square test p-value** |
| --- | --- | --- | --- | --- |
|  |  | **PCPro negative** | **PCPro positive** |  |
| **Statin use*** | **Statins used** | 46 | 25 | 0.4 |
|  | **No statins** | 16 | 13 |  |

*Statin use unavailable for 5 patients (all PCPro negative)

**Table S11.6.2 Univariable and bivariable Cox regression of statin use and PCPro in the Discovery cohort**

| **Variable*** | **Univariable Cox regression** | | **Bivariable Cox regression** | |
| --- | --- | --- | --- | --- |
|  | **Hazard ratio [95% CI]** | **p-value** | **Hazard ratio [95% CI]** | **p-value** |
| **Statin (used/not used)** | 1.22 [0.75 – 1.98] | 0.4 | 1.00 [0.61 – 1.64] | >0.9 |
| **PCPro (positive/negative)** | 3.83 [2.30 – 6.37] | <0.001 | 3.83 [2.28 – 6.42] | <0.001 |

*Only included patients who had statin use data available (n = 100)

Abbreviations: CI, confidence interval

**Table S11.6.3 Prognostic model incorporating Statin use**

| **Variables entered into the Model^+^** | **Variables in final model following LASSO shrinkage** | **C-statistic** | **p value of log-rank test** |
| --- | --- | --- | --- |
| **Model :**  Cer(d18:1/18:0), Cer(d18:1/22:0), Cer(d18:1/24:0), Cer(d18:1/24:1), Cer(d20:1/24:0), Cer(d20:1/24:1), PC(16:0/16:0), total cholesterol, HDL, triglycerides, ratio of Cer(d18:1/24:0)/Cer(d18:1/24:1), statin use | Cer(d18:1/18:0),  Total cholesterol,  Triglycerides,  Cer(d18:1/24:0)/  Cer(d18:1/24:1) | Model created  identical to final PCPro model |  |

Abbreviations: C-statistic, concordance; Cer, Ceramide; PC, phosphatidylcholine, HDL, high-density lipoprotein

Statin use was not available for the Validation cohort.

# Reference List

[1] Lin H-M, Mahon KL, Weir JM, Mundra PA, Spielman C, Briscoe K, et al. A distinct plasma lipid signature associated with poor prognosis in castration-resistant prostate cancer. International Journal of Cancer. 2017;141:2112-20.

[2] Lin H-M, Yeung N, Hastings JF, Croucher DR, Huynh K, Meikle TG, et al. Relationship between Circulating Lipids and Cytokines in Metastatic Castration-Resistant Prostate Cancer. Cancers (Basel). 2021;13:4964.

[3] Thompson SScbM. Standard additions: myth and reality. AMC technical briefs. 2009;37:1-2.

[4] CLSI. Liquid Chromatography-Mass Spectrometry Methods; Approved Guideline. CLSI document C62-A. Wayne, PA: Clinical and Laboratory Standards Institute; 2014.

[5] Lin H-M, Huynh K, Kohli M, Tan W, Azad AA, Yeung N, et al. Aberrations in circulating ceramide levels are associated with poor clinical outcomes across localised and metastatic prostate cancer. Prostate Cancer and Prostatic Diseases. 2021;24:860-70.

[6] Lin H-M, Mak B, Yeung N, Huynh K, Meikle TG, Mellett NA, et al. Overcoming enzalutamide resistance in metastatic prostate cancer by targeting sphingosine kinase. EBioMedicine. 2021;72.

[7] Weir JM, Wong G, Barlow CK, Greeve MA, Kowalczyk A, Almasy L, et al. Plasma lipid profiling in a large population-based cohort. Journal of lipid research. 2013;54:2898-908.

[8] Huynh K, Barlow CK, Jayawardana KS, Weir JM, Mellett NA, Cinel M, et al. High-Throughput Plasma Lipidomics: Detailed Mapping of the Associations with Cardiometabolic Risk Factors. Cell Chemical Biology. 2019;26:71-84.e4.

[9] Halabi S, Lin C-Y, Kelly WK, Fizazi KS, Moul JW, Kaplan EB, et al. Updated prognostic model for predicting overall survival in first-line chemotherapy for patients with metastatic castration-resistant prostate cancer. J Clin Oncol. 2014;32:671-7.
